# Complex genomic patterns of abasic sites in mammalian DNA revealed by a high-resolution SSiNGLe-AP method

**DOI:** 10.1038/s41467-022-33594-1

**Published:** 2022-10-05

**Authors:** Ye Cai, Huifen Cao, Fang Wang, Yufei Zhang, Philipp Kapranov

**Affiliations:** Institute of Genomics, School of Medicine, Huaqiao University, 668 Jimei Road, 361021 Xiamen, China

**Keywords:** Genomic analysis, DNA damage and repair, Ageing

## Abstract

DNA damage plays a critical role in biology and diseases; however, how different types of DNA lesions affect cellular functions is far from clear mostly due to the paucity of high-resolution methods that can map their locations in complex genomes, such as those of mammals. Here, we present the development and validation of SSiNGLe-AP method, which can map a common type of DNA damage, abasic (AP) sites, in a genome-wide and high-resolution manner. We apply this method to six different tissues of mice with different ages and human cancer cell lines. We find a nonrandom distribution of AP sites in the mammalian genome that exhibits dynamic enrichment at specific genomic locations, including single-nucleotide hotspots, and is significantly influenced by gene expression, age and tissue type in particular. Overall, these results suggest that we are only starting to understand the true complexities in the genomic patterns of DNA damage.

## Abstract (web summary)

Abasic (AP) sites represent a prominent type of DNA damage, yet the genomics of this lesion remains unexplored. Here, the authors report a method to map such sites at the nucleotide level in complex genomes and use it to extract complex age- and tissue-dependent patterns of AP sites in mammals.

## Introduction

DNA damage occurs continuously in every cell of an organism due to the effects of various endogenous or exogenous factors^1^. This phenomenon is manifested by multiple types of structural or chemical lesions to DNA molecules, including breaks in DNA strands, loss or chemical modifications of bases, and other types of lesions that affect the physical and chemical integrity of nascent DNA chains^2^. Due to the central role of DNA as the information repository of a cell, DNA damage and the cellular response to DNA damage are fundamentally important for basic science and human health^3^. Furthermore, DNA damage plays a central role in most of the major theories of aging^4^. In this respect, abasic (AP) sites represent one of the most common types of DNA damage, and an estimated 50,000–200,000 AP sites are present in a mammalian cell^5^. Unrepaired AP sites can block DNA replication and transcription and cause mutations^6^. In addition, a number of studies have linked unrepaired AP sites to a variety of human diseases^7^.

Three major mechanisms that lead to AP site formation are known^6^. First, various types of damaged and modified DNA bases are recognized and removed by special cellular enzymes, DNA glycosylases, which belong to the base excision repair (BER) pathway. The first step of this repair pathway involves removing the damaged base and generating an AP site in DNA^8–10^. Second, AP sites are caused by spontaneous hydrolysis of the *N*-glycosyl bond in normal bases, and this process is much more common in purines than other bases^11,12^. Third, base damage (e.g., alkylation or oxidation) can significantly destabilize the *N*-glycosylic bond and directly lead to AP site formation^6^.

Mapping sites of various types of DNA damage genome-wide and with nucleotide-level resolution is key to understanding the molecular effects of DNA damage on the cell, and method development is crucial in this endeavor. For example, there are at least 8 methods that can map double-strand breaks (DSBs) genome-wide with nucleotide-level precision, such as BLESS^13^, BLISS^14^, END-seq^15^, DSB-capture^16^ and others^17^. In contrast, there are currently only two methods that map AP sites in a mammalian genome, AP-seq^18^ and snAP-seq^19^, and only the latter has nucleotide-level precision. However, snAP-seq relies on a custom probe and complex organic chemistry^19^ that likely hampers its applicability in an average molecular biological laboratory. Moreover, both methods could detect other modifications in DNA that contain aldehyde groups. Furthermore, and even more importantly, any method likely involves a certain degree of bias, and the availability of different methods certainly increases the true understanding of genomic features of AP sites (or any other type of DNA lesion).

Therefore, in this work, we develop and extensively validate a method called SSiNGLe-AP to map AP sites, and SSiNGLe-AP is based on SSiNGLe, a method previously developed by our group to map single-strand breaks (SSBs) genome-wide with nucleotide-level resolution^20^. SSiNGLe-AP relies on a commercially available enzyme, apurinic/apyrimidinic endonuclease (APE1), making it a very practical molecular biological technique. We apply SSiNGLe-AP to generate genome-wide profiles of AP sites in 6 mouse tissue types isolated from animals with different ages and in human cancer cell lines. We find that AP site distribution in a mammalian genome is dynamic and nonrandom. For example, AP sites are more likely to be found at specific genomic elements, such as exons, promoters, and enhancers, as well as at positions corresponding to sequence variants. AP sites are enriched at specific nucleotide positions in the genome, which is defined in this work as AP site hotspots, that can be partially generated via BER and strongly associate with certain regulatory elements. Genomic patterns of AP sites are significantly influenced by gene expression, age and especially by tissue type. Overall, our data suggest that the distribution of DNA damage in the genome is very complex and that we are only starting to understand the true genomic complexities of this phenomenon.

## Results

### Development and initial validation of SSiNGLe-AP

**Fig. 1:**
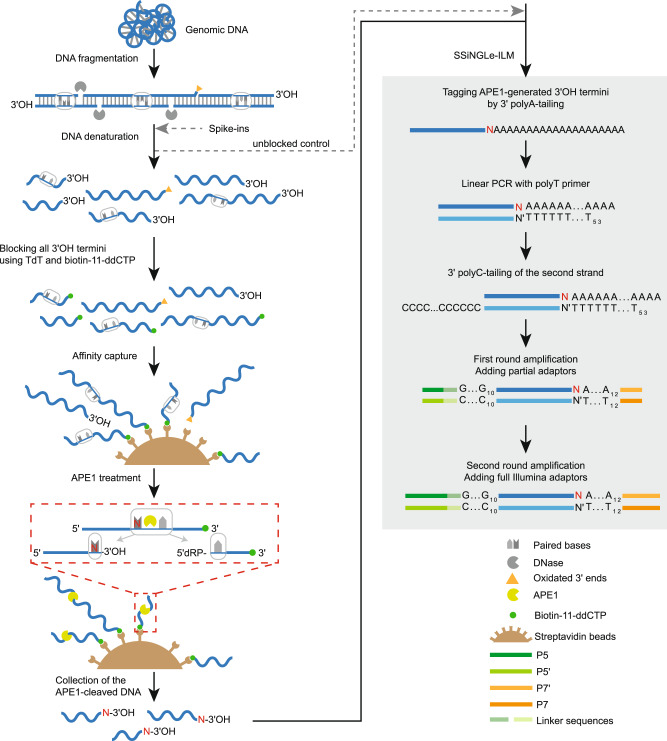
Schematics of SSiNGLe-AP. Workflow of the molecular biological part of SSiNGLe-AP starting with fragmentation of genomic dsDNA using DNase, followed by denaturation, blocking of all 3’OH termini of ssDNA molecules using TdT (terminal transferase) and biotin-11-ddCTP, and subsequent affinity capture of 3’-blocked and biotinylated fragments (all symbols are explained in bottom right) that were then subjected to APE1 treatment that releases ssDNA fragments, in which the 3’OH termini correspond to Ns—the nucleotides immediately upstream of the corresponding AP sites. The freed fragments were then subjected to the SSiNGLe for Illumina (SSiNGLe-ILM) procedure starting with tagging the 3’OH termini by polyA-tailing and subsequent NGS library preparation steps. Importantly, DNA molecules in which the 3’ termini were blocked by DNA damage (such as oxidated 3’ ends) cannot be biotinylated and thus did not enter the APE1 treatment step. The spike-ins were added to DNA before denaturation, as shown. To generate the unblocked control, the denatured DNA was used as input into SSiNGLe-ILM, as shown after the addition of spike-ins. See Supplementary Fig. 1 for the description of the analytical parts of SSiNGLe-AP.

AP sites can be converted into SSBs by the commercially available enzyme APE1, which belongs to the BER pathway and catalyzes endonucleolytic cleavage of the phosphodiester bond immediately 5’ to the AP sites^21^. The resulting SSBs containing a 3’OH terminus were then mapped using the single-strand break mapping at the nucleotide genome level (SSiNGLe) method, which was developed previously by our group^20^; thus, the position of the AP sites were revealed (Fig. 1). However, endogenous SSBs also represent a very common type of DNA lesion^22^; thus, if not blocked before APE1 treatment, SSBs could generate a significant background. Therefore, in our method, purified genomic DNA is fragmented by DNase to 1000–2500 bp, denatured and then breaks caused by DNase fragmentation, and all endogenous breaks with 3’OH termini are blocked using terminal transferase (TdT) and biotinylated-ddCTP (Fig. 1). The blocked DNA is then captured using streptavidin-coated magnetic beads. Following a series of washes to remove the unbound and thus unblocked DNA, the blocked DNA bound to the beads is treated with APE1 enzyme that can cleave AP sites in both double- and single-stranded DNA^23^ (Fig. 1). The liberated, cleaved DNA is then used as an input into the SSiNGLe-ILM protocol—a SSiNGLe protocol developed for Illumina platform^20^ that will simply be referred to as SSiNGLe below (Fig. 1).

Briefly, as illustrated in Fig. 1, the 3’OH termini created by cleavage with APE1 were polyA-tailed using TdT, blocked and extended with an oligo-dT primer. The extended DNA strands were then polyC-tailed and amplified using PCR primers containing polyG and polyT sequences. Following paired-end next-generation sequencing (NGS) using the Illumina platform of the libraries from APE1-treated DNA, the pairs of reads starting with both polyG and polyT sequences that corresponded to the original polyC and polyA tails were mapped to the genome. PCR duplicates were then removed and only uniquely aligning reads were selected for the downstream analyses. The positions of the original APE1-generated 3’OH termini were inferred based on the first aligned positions of the reads starting with the polyT sequences. To avoid reads obtained by priming in the endogenous genomic polyA sequences, reads mapping adjacent to such sequences were removed as performed in the previously published SSiNGLe protocol^20^. Finally, the positions of the corresponding AP sites were assigned by shifting the coordinates of the APE1-generated termini by one base (Supplementary Fig. 1a).

**Fig. 2:**
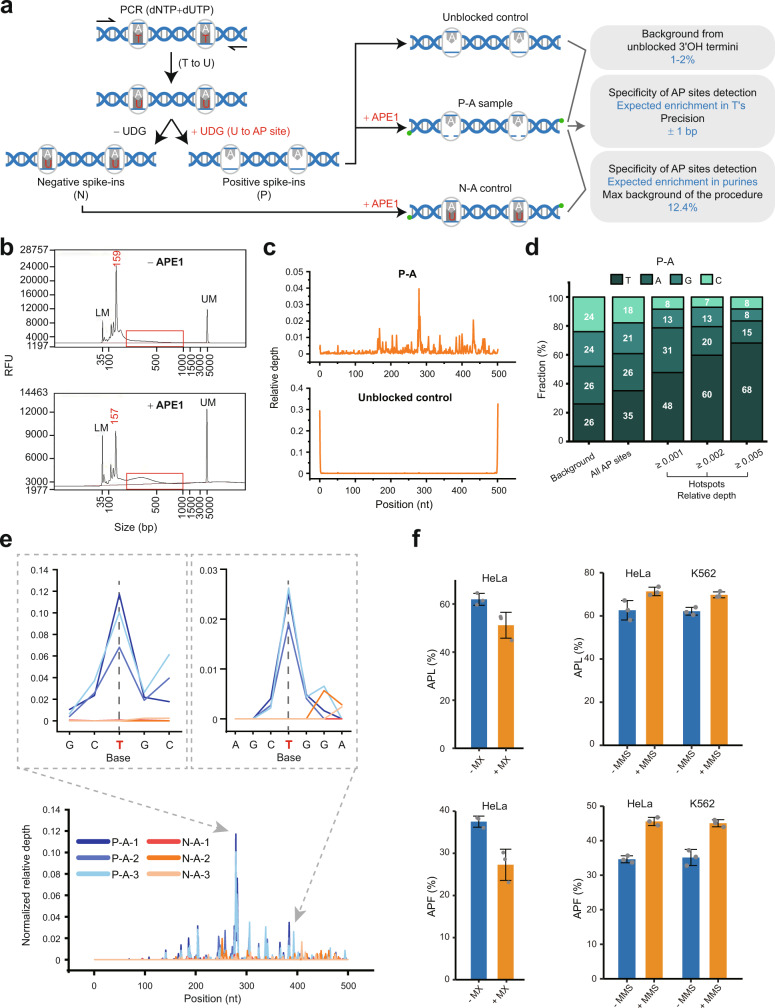
Validation of SSiNGLe-AP. **a** Preparation and application of the spike-ins. The spike-ins were PCR amplified in the presence of dUTP and then either treated with UDG (uracil-DNA glycosylase) to produce AP sites at thymines (Ts) and serve as the positive spike-ins (“P”) or were not treated and serve as the negative spike-ins (“N”). Mammalian DNA samples containing the positive and negative spike-ins were processed by the SSiNGLe-AP procedure to generate the “P-A” samples, “N-A” or unblocked (Fig. 1) controls and used to estimate the background, specificity and precision of the method. **b** Representative DNA size distribution profiles of NGS libraries made using SSiNGLe-AP with (“+APE1”) or without (“‒APE1”) the APE1 treatment from mammalian DNA containing the positive spike-ins. The portions of the profiles representing the actual genomic material are boxed—the peaks to the left represent primer dimers. **c** Aggregate plots of relative depths of AP sites (“Methods”) along the length of positive spike-in sequences in the “P-A” or unblocked controls generated by combining sites from both strands of all spike-ins and all technical replicates. **d** Bar plots indicate the average fractions of each nucleotide occurring in the spike-in sequences (“Background”), as well as in all AP sites and hotspots detected with variable thresholds in the 3 spike-ins of the “P-A” samples (“Methods”). **e** Normalized relative depth (Methods) of mapped sites from 3 independent technical replicates of positive (“P-A”) and negative (“N-A”) spike-ins are plotted along the length of the “+” strand of the spike-in S1. Zoomed-in windows show two AP site hotspots, marked by arrows, peaking at T nucleotides. **f** The abundancies of AP sites in the samples treated with MX (methoxyamine), MMS (methyl methanesulfonate) and the corresponding controls are represented as either APL or APF (“Methods”). Data are presented as mean values +/− SD based on 3 biological replicates. Source data are provided as a Source data file.

To evaluate the performance of the method, we prepared 3 bacterial DNA fragments containing AP sites and spiked them into the background of human genomic DNA after the fragmentation (Supplementary Fig. 2). To generate AP sites in the spike-ins (Fig. 2a), first, uracil was incorporated into the bacterial control sequences by PCR in the presence of 1:125 dUTP:dTTP. Second, the PCR products were treated with uracil-DNA glycosylase (UDG)—an enzyme that recognizes uracil bases in DNA and converts them into AP sites^24^—and served as the positive spike-ins, which should contain AP sites at the nucleotide positions where thymines were present in the original sequences. However, the UDG-generated AP sites are likely not the only source of abasic sites in the spike-in sequences since AP sites can also occur by spontaneous depurination of DNA^11,12^, which is aided by the high-temperature (94 °C) exposure of DNA during the PCR process. Therefore, to investigate the contribution of such artifactual sites, we prepared negative spike-ins that contained uracil but were not treated with UDG (Fig. 2a)—such spike-ins are expected to contain only spontaneous and PCR-induced AP sites. The positive and negative spike-ins were then added to the human genomic DNA before the denaturation step in the SSiNGLe-AP procedure, as shown in Fig. 1. Each such experiment was performed in 3 independent technical replicates.

In addition, the DNA samples containing the positive spike-ins were used to generate 2 additional controls. First, the APE1 treatment was omitted in the SSiNGLe-AP procedure—since AP sites can be detected only after APE1 cleavage, this “−APE1” control should estimate the signal derived from the abasic sites. Second, fragmented DNA was used as input into the polyA-tailing procedure that bypassed the blocking, bead selection and APE1 treatment steps. Such samples will be referred to as the “unblocked” controls (Fig. 1), and used to estimate (1) the blocking efficiency and (2) the overall technical bias of the method (see below).

As shown in Fig. 2b, libraries prepared from the DNA treated with APE1 had a significantly higher signal compared to that of the “−APE1” control, thus strongly suggesting that the vast majority of the signal in the SSiNGLe-AP procedure should be derived from APE1 cleavage and, by inference, AP sites. The distribution of reads mapping to spike-in sequences was then analyzed to estimate the following properties of the method. First, the background from the free 3’OH termini failed to be blocked. As illustrated in Fig. 2c, the background was very low (1–2%), as evidenced by the very strong enrichment in the signal at the 3’ ends of the spike-ins in the unblocked control that virtually disappeared in the blocked APE1-treated samples (Supplementary Data 1 and 2). Second, true AP sites were detected. As expected, the nucleotides corresponding to the AP sites found in the positive spike-ins were enriched at thymines, and the enrichment increased with increasing the relative depth of detection for the AP sites (Fig. 2d and Supplementary Data 3, see “Methods: Spike-in analysis”). Third, the detection precision was estimated by the profiles of peaks for the spike-in AP sites that clearly showed enrichment at specific thymine positions, and most of the signal were within 1 nucleotide from the T base (Fig. 2e and Supplementary Data 4).

Among the nucleotides corresponding to the nonthymine AP sites in the positive spike-ins, purines (A’s and G’s) were dominant (Fig. 2d) and thus also likely represented the AP sites generated in these spike-ins during preparation as mentioned above. To further estimate the contribution of artifactual AP sites, we compared the signal in the positive and negative spike-ins. As shown in Fig. 2e, the magnitude of the signal was much higher in the former than in the latter. Sites in the negative spike-ins are derived from the (1) unblocked 3’ ends and (2) artificial AP sites produced by the sample preparation. The median fractions of the AP sites derived from both sources, or just the latter one, were estimated to be 12.4% and 11.5%, respectively (Supplementary Data 1). However, these estimates provide the maximum bound of the background in our assays because the exposure of genomic DNA to high temperature is much shorter than that with the spike-ins. The contribution of artifactual depurination of genomic DNA during the actual SSiNGLe-AP can only occur before the polyA addition step (Fig. 1), and the total time during which DNA is exposed to 95 °C before that step is 1 min compared to the 18 min exposure used in the spike-in preparation procedure. Therefore, the true background in SSiNGLe-AP, at least based on the spike-in experiments, should be closer to the background from the unblocked sites that was estimated to be 1–2%, as mentioned above (also see below). In summary, the results from the spike-in experiments showed that SSiNGLe-AP can detect AP sites with low background and nucleotide level precision.

### Further validation of the performance of SSiNGLe-AP on mammalian genomes

To further validate the performance of SSiNGLe-AP in the detection of true endogenous AP sites in mammalian genomes, we tested two conditions that decreased or increased the number of detected AP sites (Supplementary Fig. 2). First, we treated DNA isolated from human HeLa cells in vitro with methoxyamine (MX), which can react with the aldehyde of an abasic site, making it resistant to cleavage by APE1^25^. Second, we treated HeLa and K562 cells in vivo with the alkylating agent methyl methanesulfonate (MMS) which is well known to induce AP site formation^26^ (also see below). If SSiNGLe-AP indeed measures true AP sites, we should expect a global decrease in sites detected in the MX-treated DNA and an increase in the MMS-treated samples.

To evaluate the treatment effect on the total amount of AP sites, we calculated 2 metrics. First, based on the size distribution of the sequencing libraries, we could estimate the fraction of DNA molecules that are larger than primer dimers and therefore should represent the amplification products derived from the genome, which we will refer to as APL (Supplementary Fig. 3a, see “Methods: Calculation of the abundances of AP sites and SSBs”). Second, after sequencing, we estimated the fraction of reads corresponding to AP sites out of the total reads that started with the polyA and polyC stretches and defined it as APF (Supplementary Fig. 1a).

As shown in Fig. 2f, both metrics consistently showed statistically significant trends in the expected directions for both MX- and MMS-treated samples (*p*-values <0.05, one-sided *t* test, Supplementary Data 5). The MX treatment resulted in 17.4% and 27.3% (average) drops in the fraction of AP sites based on the APL and APF, respectively (Fig. 2f and Supplementary Data 5). Reciprocally, the MMS treatment resulted in average increases of 13.9% and 31.6% based on APL and APF, respectively, in HeLa cells, with corresponding values of 12.3% and 28.3% for K562 cells (Fig. 2f and Supplementary Data 5).

We then characterized the complexity of the libraries generated using SSiNGLe-AP and the technical reproducibility for the detection of AP sites in complex mammalian genomes by generating two separate libraries from four samples, which were heart and liver tissues from 3- and 19-month-old C57BL/6J mice, and deep-sequencing them at 10-fold higher scales compared to most of the other samples (Supplementary Data 6 and Supplementary Fig. 2). The reason for choosing these two tissue types was to assess the reproducibility in samples that had high (heart) and low (liver) estimated abundances of AP sites (see below). As shown in Supplementary Fig. 3b, the discovery of AP sites was far from plateauing in most of the libraries, especially those from the heart, which was consistent with the high complexity of these libraries and the high abundance of AP sites in mammalian cells^5^. For each pair of technical replicates, we separately assessed reproducibility for the AP sites detected in the whole genome by variable depths of at least 1, 2, 3, 4, or 5 reads in both technical replicates (Supplementary Fig. 3c and Supplementary Data 7). The reproducibility was defined as the odds ratio of overlap, and a ratio of 1 represented a random overlap (see Methods: ‘Calculations of the odds ratios of enrichment of all AP sites or hotspots for the different overlap analyses’). The observed odds ratios ranged from 3.1 to 3.7 for all sites (detected by ≥1 read) (Supplementary Fig. 3c and Supplemental Data 7). There was, however, a striking increase in the significance of overlap with the increase in the minimal depth required to detect an AP site in both replicates (Supplementary Fig. 3c and Supplementary Data 7). All the odds ratios were statistically significant, as estimated by the two-sided binomial test, and the reproducibility patterns were quite similar for both tissues (Supplementary Data 7 and Supplementary Fig. 3c). These results suggest that SSiNGLe-AP can reproducibly detect AP sites in mammalian genomes, and this reproducibility increases for the single-nucleotide AP sites detected by multiple reads, which we will define as hotspots of AP sites. Moreover, we also observed high technical reproducibility of enrichment of AP sites in various genomic elements (Supplementary Fig. 3d, e and Supplementary Data 6).

To estimate the contribution of the potential bias at various steps in the SSiNGLe-AP procedure to the distribution of AP sites, we generated the “unblocked” control (Fig. 1, also see above) for each deeply sequenced sample above and sequenced it at the same scale (Supplementary Data 6). The “unblocked” control has many steps in common with the actual SSiNGLe-AP procedure, such as DNase fragmentation, polyA-tailing, PCR and the analysis pipeline, with the exception of specific steps related to AP site detection (Fig. 1 and Supplementary Fig. 1a). We then estimate the variances attributable to the method itself (see Methods: ‘Estimation of the technical bias of the method’) for all AP sites or the sites detected by at least 2 reads in the nonrepeat regions of the genome by the SSiNGLe-AP procedure as respectively 2% and 5% across all 8 samples. While both estimates are significant with *p*-values <2.2E−16, which suggests that some bias does exist, they also demonstrate that the contribution of bias to the outcome of the SSiNGLe-AP procedure is small. However, the corresponding variances increased to 5% and 12% and were still statistically significant when the repeats were included, possibly due to the bias introduced at the read alignment stage, which is more challenging for repeated sequences. Thus, even though the contribution of the bias was still relatively small, unless indicated otherwise, all analyses below were conducted on the nonrepeat regions of the genome.

Since APE1 could also involve bias in terms of the preferential cleavage of AP sites in a certain sequence context, we performed the SSiNGLe-AP procedure using a nonhomologous AP endonuclease—*E. coli* Endonuclease IV (Endo IV)^27^ (Supplementary Figs. 2 and 4a), which can also cleave single-stranded DNA^28^—on the DNA from K562 and 4 mouse samples. We then calculated the odds ratios of overlaps between the AP sites detected by at least 1, 2, 3, 4 or 5 reads anywhere in the genome, including repeats, in the APE1 and Endo IV experiments. As shown in Supplementary Fig. 4b, c, the overlaps between the treatments were highly significant (Supplementary Data 8). The odds ratios for sites detected by ≥1 read were in the range of 4.6–9.2 and then increased significantly with the increase in the depth of detection; for example, the corresponding ranges for sites detected by ≥2 or ≥3 reads were from 477.2 to 7951.2 and from 72,839.2 to 4,975,750.4, respectively (Supplementary Fig. 4b, c and Supplementary Data 8). Therefore, the nonhomologous AP endonuclease Endo IV had a strong tendency to detect AP sites at the same locations as that of APE1, and that tendency increased significantly for the sites detected by multiple reads (also see below), further validating the true nature of the detected AP sites and the reproducibility of the method.

Altogether, the results presented above suggest that SSiNGLe-AP can detect true AP sites in complex mammalian genomes with high resolution and reproducibility and low background and technical bias.

### Dynamic landscape of AP sites in the mammalian genome

We then investigated the general hallmark features of the genomic profiles of AP sites, as well as the effects of tissue types and age on these features by applying SSiNGLe-AP to DNA extracted from 6 mouse tissues and cell types—bone marrow, brain, heart, liver, peripheral blood mononuclear cells (PBMCs) and sperm—that were isolated from 3-, 12-, 19-, and 22-month-old animals (Supplementary Fig. 2). Each tissue/cell type and age combination was represented by samples extracted from 3 different animals from the inbred C57BL/6J background for a total of 72 samples. Each genomic DNA sample contained the bacterial spike-in sequences to control for the efficiency of blocking of the endogenous breaks. With the exception of one sample, all other 71 samples had a median background from unblocked termini of 1.1% (from 0.3% to 4.5%) and were used for the downstream analysis (Supplementary Data 9).

**Fig. 3:**
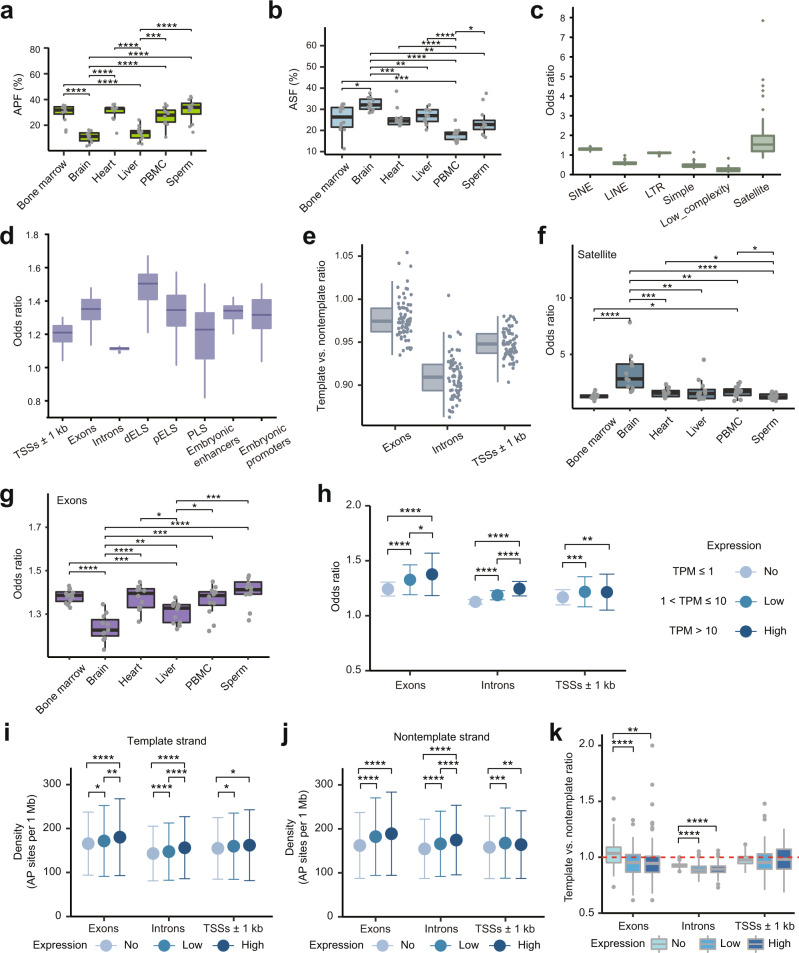
Landscape of all AP sites in the mouse genome. **a** Global levels of AP sites. **b** Global levels of SSBs. **c** Overlap between AP sites and repeats. **d** Overlap between AP sites and genomic elements. The dELS, pELS, and PLS represent candidate *cis*-regulatory elements with distal enhancer-like, proximal enhancer-like and promoter signatures, respectively. **e** Distribution of the template vs. nontemplate ratios for the AP sites found in the indicated elements. **f** Overlap between the AP sites and satellite repeats. **g** Overlap between the AP sites and exons. **h** Odds ratios of overlap between AP sites and the indicated elements. **i** Density of AP sites on the template strands of the indicated elements. **j** Density of AP sites on the nontemplate strands of the indicated elements. **k** Box plots of the template vs. nontemplate ratios for the AP sites located at the indicated elements. Genes were stratified by expression level in each sample, and the overlap with the AP sites was calculated for each stratum in each sample (**h**–**k**). Data are presented as mean values +/− SD (**h**–**j**). Overlaps between AP sites and the indicated elements are shown as box plots of the odds ratios (**c**, **d**, **f**, **g**). Box plots indicate median (middle line), 25th, 75th percentile (box) and 1.5× interquartile range (whiskers) as well as each individual data (single points, **a**, **b**, **e**–**g**) or outliers (single points, **c**, **d**, **k**). Data based on 12 (3 biological replicates of 4 age groups) biologically independent samples per tissue type with the exception of brain represented by 11 samples (**a**, **b**, **f**, **g**), or all 71 mouse samples (**c**–**e**, **h**–**k**) are shown. Asterisks above connecting lines indicate significance of differences between the indicated pairs of tissues (**a**, **b**, **f**, **g**) or gene expression categories (**h**–**k**) as represented by raw *p*-values of ≤0.05 (*), ≤0.01 (**), ≤0.001 (***), and ≤0.0001 (****) calculated by the two-sided Wilcoxon rank-sum test (**a**, **b**, **f**, **g**) or the two-sided Wilcoxon signed rank test (**h**–**k**). Source data are provided as a Source data file.

Of the ~3.4 million (median) quality-filtered read pairs obtained per sample, after the various stages of filtering (Supplementary Fig. 1a), we obtained a median of 0.65 million reads that represented AP sites per sample (Supplementary Data 9). We first explored the distribution of the global amount of AP sites in the different samples. Furthermore, since the AP sites were converted into SSBs with 3’OH termini via APE1 during the normal repair process, we also estimated the abundance of SSBs in the different tissues by profiling the latter in the same genomic DNA preparations used to detect AP sites from the 71 samples (Supplementary Fig. 2). Interestingly, the estimates of AP sites based on the APF metric were quite consistent among the different mouse samples of the same tissue type (Fig. 3a and Supplementary Data 9). Strikingly, the brain and liver had significantly lower APFs (median APFs of 11.4% and 14.5%, respectively) than those of the other four tissues (median APF of 31.9%), as shown in Fig. 3a (Supplementary Data 9). These results suggest that the brain and liver have significantly lower amounts of AP sites than that in the other tissues.

We then calculated the corresponding fractions of reads representing SSBs, using the ASF metric (see “Methods: Calculation of the abundances of AP sites and SSBs”), across the 71 samples (Supplementary Data 10). Strikingly, the two tissues that had the lowest fraction of AP sites, the brain and liver, had higher ASFs than those of the other four tissues, and the brain contained the highest fraction (Fig. 3b and Supplementary Data 10). These results have two implications. The first implication, which is technical, is based on the observation that the tissue with the highest fraction of SSBs does not have the highest fraction of AP sites, which is consistent with the high efficiency of blocking endogenous 3’OH termini that was reported above. The second implication is biological and, while not definitive, is at least consistent with the notion that the tissue-specific differences in the amount of AP sites could be explained by the efficiency of the repair system, which converts the sites into SSBs (also see below). AP sites and SSBs are considered to represent two of the most abundant types of DNA lesions in a mammalian genome^5,22^. Our results suggest that depending on the tissue type, the relative abundances of these two types of DNA damage differ.

Approximately one-third (37.2%, median) of the AP sites mapped to repetitive elements in each sample (Supplementary Data 9). However, the AP sites differed in relative enrichment in various types of repeats as measured by the odds ratio of enrichment over the expected values by chance (Fig. 3c, Supplementary Fig. 1b, and Supplementary Data 11, see “Methods: Calculations of the odds ratios of enrichment of all AP sites or hotspots for the different overlap analyses”). Most notably, satellite repeats were most enriched, with a median odds ratio of 1.53 compared to that of all other repeat classes (Fig. 3c and Supplementary Data 11). We then explored the association of nonrepeat AP sites with various genomic elements, such as exons and introns of genes and regulatory regions (Fig. 3d, Supplementary Fig. 1b, and Supplementary Data 12). The latter were obtained from the following sources: (1) promoter regions of all genes defined as ±1 kb around transcriptional start sites (TSSs) of all annotated mouse genes^29^; (2) candidate cis-regulatory elements (cCREs) with promoter-like (PLS), proximal enhancer-like (pELS) and distal enhancer-like (dELS) signatures^30^; and (3) promoters and enhancers derived from different stages of mouse embryonic development^31^. Overall, we observed a moderate, but statistically significant, enrichment of AP sites in exons, introns and all types of regulatory regions in most samples, with median odds ratios of enrichment ranging from 1.12 to 1.50 (Fig. 3d and Supplementary Data 12).

The AP sites had a tendency to map to the nontemplate strands of genes and ±1 kb TSS regions, which was evidenced by the observation that the template vs. nontemplate ratios were mostly less than one (Fig. 3e and Supplementary Data 12). However, while still showing a preference for the nontemplate strand, this ratio was significantly higher (*p*-value 2.48E−13, Wilcoxon signed rank test) in exons than in introns, suggesting that AP sites had a higher tendency to map to the template strand in exons than in introns (Fig. 3e), which was similar to what was previously observed for SSBs^20^.

Overall, the abovementioned profiles for the enrichment in AP sites in various genomic features were quite similar to those found in the libraries sequenced at a 10-fold higher scale (Supplementary Fig. 3d and Supplementary Data 6), thus showing that the genomic profiles of AP sites were quite reproducible and robust. Moreover, the patterns of enrichment of AP sites in different genomic features and the nontemplate strand could also be observed in the Endo IV experiments, suggesting that the detected associations were not caused by the potential APE1 preference for AP sites located within certain sequence contexts (Supplementary Fig. 4d, e and Supplementary Data 12). Furthermore, since, the contribution of the bias of the other SSiNGLe-AP procedure steps to the overall signal is small, as mentioned above, the results shown here should reflect the true association of AP sites and various genomic features.

Interestingly, the strengths of the associations of AP sites in the brain and, to a lesser extent, liver with multiple types of genomic features were significantly different from those of other tissues (Fig. 3f, g, Supplementary Fig. 5, and Supplementary Data 12). The most striking difference was exhibited by higher the enrichment of brain AP sites in multiple classes of repeats, and most notably in satellite repeats (Fig. 3f). Furthermore, even when considering only nonrepeat AP sites, the brain and liver had a tendency to have significantly lower the enrichment of AP sites in exons and all types of regulatory elements (Fig. 3g and Supplementary Fig. 5f–k). On the other hand, these two tissues exhibited significantly higher template vs. nontemplate ratios in exons than other tissues, suggesting that AP sites in these tissues tend to occur more often on the template strand of exons (Supplementary Fig. 5l).

DNA damage and repair are well known to have complex interactions with transcription^32,33^. Therefore, we explored whether the transcribed status of genes has an effect on the distribution of AP sites at and around gene boundaries. To investigate this, we performed RNA-seq analysis on RNAs extracted from the same 71 samples that were used for AP profiling (Supplementary Fig. 2). We then calculated the expression level of each annotated gene using the transcripts per million (TPM) metric and used the results to stratify the genes into 3 expression categories—non, low- and high-expressed—in each of the 71 samples (see “Methods: RNA-seq analysis”). We observed a significant increase in the enrichment of AP sites in exons and introns with increasing gene expression levels (Fig. 3h and Supplementary Data 13). Significantly higher enrichment of AP sites was also present in the flanking (±1 kb) regions around TSSs of the expressed genes than those of nonexpressed genes (Fig. 3h). Strikingly, the densities of AP sites on both the template (Fig. 3i) and nontemplate (Fig. 3j) strands of exons and introns increased with increasing expression level (Supplementary Data 13). However, AP sites had the tendency to increase more on the nontemplate strands, as demonstrated by significantly lower template vs. nontemplate ratios in both the exons and introns of expressed genes (Fig. 3k and Supplementary Data 13). The TSS-flanking regions of expressed and nonexpressed genes did not reveal the strand preference of AP sites (Fig. 3k).

We also investigated whether the expression of the mouse *Apex*1 gene encoding APE1 could potentially explain the tissue-specific differences in the abundances of AP sites and SSBs (Fig. 3a, b). While no statistically significant differences could be found, we did observe that the expression of *Apex1* mRNA had a slightly higher average in the brain and liver, the two tissues with the lowest fractions of AP sites and the highest fractions of SSBs (Fig. 3a, b and Supplementary Data 9, 10, 14). Overall, the levels of *Apex1* mRNA were slightly negative (−0.17) and slightly positive (0.1) Pearson correlations with APF and ASF were obtained across the 71 samples. Therefore, while not definitive, these results suggest that the activity of various components of the DNA repair pathway might account for the differences in the abundance of these DNA lesions.

### AP sites tend to associate with sequence variants

**Fig. 4:**
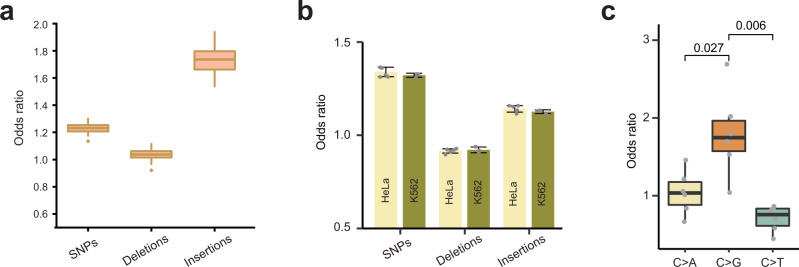
Association of AP sites with sequence variants. **a** Overlap of AP sites and sequence variants across the 71 mouse samples. **b** Odds ratios of overlap of AP sites with sequence variants in HeLa (6 biological replicates) and K562 (3 biological replicates) cell lines. Data are presented as mean values +/− SD. **c** Overlap between AP sites and the C alleles for the 3 indicated variant types found across 6 biological replicates of HeLa cell line. The numbers above connecting lines are *p*-values calculated by one-sided paired *t* test. Box plots indicate median (middle line), 25th, 75th percentile (box) and 1.5× interquartile range (whiskers) as well as outliers (single points, **a**) or each individual data (single points, **c**). Source data are provided as a Source data file.

We have previously found a significant enrichment in SSBs at locations in the human genome where sequence variants, especially single nucleotide polymorphisms (SNPs), have been detected in the human population, suggesting that unrepaired or improperly repaired SSBs could lead to mutations^20^. Therefore, we also investigated whether such an association exists between AP sites and locations in the mouse genome where SNPs, insertions or deletions have been previously found (Fig. 4a, Supplementary Fig. 5o–q, and Supplementary Data 12). As shown in Fig. 4a, we found a statistically significant association of AP sites with all 3 types of variants; however, AP sites had the strongest tendency to overlap with the positions of insertions (median odds ratio 1.74), followed by moderate association with SNPs (median odds ratio of 1.23) and very low association with deletions (median odds ratio of 1.04). Interestingly, AP sites found in sperm had a higher association with SNPs than several other tissues (Supplementary Fig. 5o). The relative enrichment among the different types of variants was very similar when the scale of sequencing was increased 10-fold (Supplementary Fig. 3e and Supplementary Data 6) or Endo IV was used instead of APE1 (Supplementary Fig. 4f and Supplementary Data 12). Interestingly, AP sites in the two human cell lines HeLa and K562 were also enriched at sites of human sequence variation (Fig. 4b and Supplementary Data 5). However, in both cell lines, AP sites had the highest association with SNPs, followed by insertions, but had no association with deletions (Fig. 4b).

AP sites are highly mutagenic^6^ and are believed to be responsible for the C > G mutation signature found in a number of cancers^34^ due to the combined action of the following cellular processes: (1) deamination of cytosines by the APOBEC3 family of enzymes, resulting in uracils^35^, which are then repaired by BER via generation of AP sites; and (2) error-prone translesion synthesis (TLS) across the unrepaired AP sites^36^. Since one of the cancer types that exhibits the C > G signature was cervical cancer^34^, which is represented by the HeLa cell line used in this work, we explored whether the AP sites detected in this cell type using SSiNGLe-AP were associated with the C > G mutations (Supplementary Fig. 2). To address this, we resequenced the genome of our HeLa clone and identified C > G, C > A and C > T variants in the TpCpN context that are favored by APOBEC3 cytosine deaminases^37^. For this analysis, we focused only on heterozygous SNPs, in which one of the alleles was represented by a cytosine that could still be deaminated by APOBEC3. We then estimated the odds ratios for the overlap of the C alleles for each of the 3 variant types (C > G, C > A or C > T) with HeLa AP sites in 6 biological replicates (see “Methods: Genome resequencing of HeLa cell line“). Indeed, we found that the odds ratios for the C > G variants overlapping AP sites were significantly higher than the corresponding ratios for the other 2 variant types in all 6 bioreplicates (Fig. 4c and Supplementary Data 5).

### Existence and properties of hotspots of AP sites

**Fig. 5:**
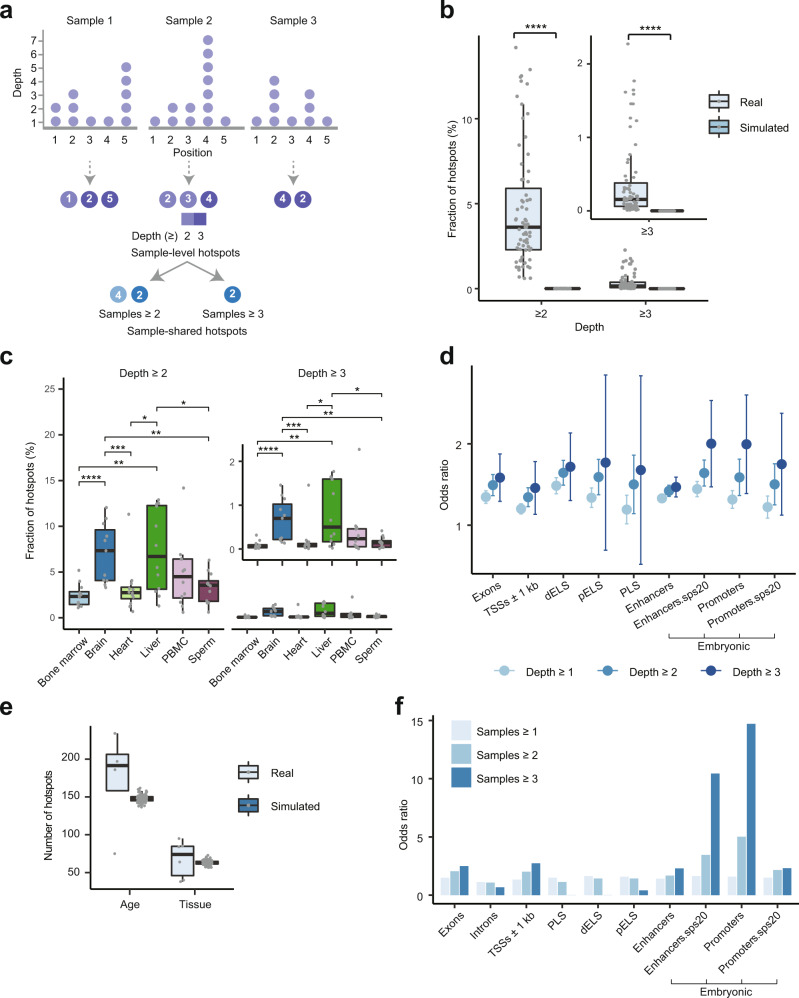
Properties of AP site hotspots in mouse genome. **a** Illustration of the analytical steps used to define sample-level and sample-shared hotspots. **b** Comparison of the fraction of sample-level hotspots found in the real and simulated datasets at different read depths across the 71 mouse samples. **c** Comparison of the fractions of sample-level hotspots among different tissues (*X*-axes) at different read depths. Data based on 12 (3 biological replicates of 4 age groups) samples per tissue type (with the exception of 11 for brain) are reported. **d** Dot plots show the increase in the odds ratios of association between the sample-level hotspots detected with the indicated read depths and different genomic elements. Data are presented as the mean values +/− SD based on the 71 mouse samples. **e** Box plot of the numbers of age- or tissue-specific sample-shared hotspots found in the real and simulated datasets. Data based on the 4 age groups or 6 tissue types in the real datasets, and 100 corresponding random simulations are shown. **b**, **c**, **e** Box plots indicate median (middle line), 25th, 75th percentile (box) and 1.5× interquartile range (whiskers) as well as each individual data (single points). **f** Odds ratios of association between all hotspots (“Samples ≥1”) or just sample-shared hotspots (“Samples ≥2” and “Samples ≥3”) and various genomic elements (*X*-axis). The dELS, pELS, and PLS represent candidate *cis*-regulatory elements with distal enhancer-like, proximal enhancer-like and promoter signatures, respectively, and the “sps20” in a feature name means that the features were found in at least 20 embryonic tissues and/or times of development (**d**, **f**). Asterisks above connecting lines indicate significance of difference between the real and simulated datasets (**b**) or indicated pairs of tissues (**c**) as represented by raw *p*-values of ≤0.05 (*), ≤0.01 (**), ≤0.001 (***) and ≤0.0001 (****) calculated by the two-sided paired *t* test (**b**) or two-sided Wilcoxon rank-sum test (**c**). Source data are provided as a Source data file.

As shown in the analyses above, AP sites detected by multiple reads had a far greater chance of being detected in different technical replicates and by different AP endonucleases (APE1 and Endo IV) than sites detected by a single read. Therefore, we tested whether the occurrence of the former could be explained purely by chance. For each of the 71 mouse samples, we identified the genomic positions of AP sites detected by at least 2 or 3 reads (Fig. 5a and Supplementary Data 15). Then, for every sample, we repeated the procedure, but utilized a simulated random AP dataset that was generated with the same number of reads. As shown in Fig. 5b, the numbers of sites in the simulated datasets were significantly lower at both read depths compared to the real datasets (Supplementary Data 15). These results suggested that AP sites detected by multiple reads cannot be explained by random chance but rather could represent nucleotides in the genome where AP sites are more likely to occur—which are called AP site hotspots.

Most of these sites were sample specific; 1,036,139 (99.7%) out of 1,038,844 hotspots with a depth of ≥2 reads were found in only one sample. However, since the detection of hotspots could be influenced by sequencing depth, we investigated whether the high sample specificity of their detection could have some biological foundation. We calculated the fraction of hotspots compared to all AP sites per sample and found that samples from the brain and liver had a tendency to exhibit higher fractions of hotspots relative to those of other tissues (Fig. 5c and Supplementary Data 15). Since the brain and liver had the smallest numbers of total AP sites (Fig. 3a, see above), the differences in the numbers of detected sites therefore cannot be the only reason for the sample-specific nature of the hotspots (also see below). Interestingly, sample-level hotspots had a tendency to have a higher association with functional genomic elements, such as exons and regulatory regions, as evidenced by a progressive increase in the odds ratios of the overlaps between the hotspots and these elements with increasing hotspot depth (Fig. 5d and Supplementary Data 12 and 16). Moreover, sample-level hotspots exhibited stronger enrichments at exons, introns and TSS-flanking regions with increasing expression levels (Supplementary Fig. 6a and Supplementary Data 13). However, the enrichment of hotspots was always higher than that of all AP sites at each genomic element and at each expression level. (Supplementary Fig. 6a and Supplementary Data 13). Finally, similar to all AP sites, the hotspots were also more enriched on the nontemplate strands of exons and introns with increased expression (Supplementary Fig. 6b and Supplementary Data 13).

Intrigued by the higher tendency of hotspots to occur in the functional elements, we further investigated this phenomenon by exploring the hotspots shared by multiple samples. Such sample-shared hotspots represented a minority of the 1,038,844 hotspots with a depth of ≥2: 2705 (0.26%) and 231 (0.02%) of such hotspots were shared by at least 2 or 3 samples, respectively (Supplementary Data 17). To determine if samples sharing certain biological features, such as tissue type or age, had more hotspots in common, sample labels were randomly permuted, the number of hotspots shared by different numbers of samples was calculated, and the process was repeated 100 times. Indeed, the number of hotspots shared by samples derived from the same tissue or age group was always higher (Fig. 5e).

Strikingly, the sample-shared hotspots had a very strong tendency to be enriched in embryonic promoters and constitutive embryonic enhancers found in multiple embryonic tissues and/or times of development. For example, the corresponding odds ratios of enrichment for the embryonic promoters and constitutive enhancers were 14.71 and 2.30 for the hotspots shared by 3 samples (Fig. 5f and Supplementary Data 17). Examples of the sample-shared hotspots mapping to constitutive enhancers are shown in Supplementary Fig. 7a.

On the other hand, there was a strong drop in association with cCREs (PLS, dELS and pELS), as shown in Fig. 5f. The reason for the preferential enrichment of the sample-shared hotspots in one type of regulatory element and depletion in another, which was not observed with the sample-level hotspots (Fig. 5d), is not clear. The samples used to map the embryonic promoters/enhancers were included in those used to map cCREs by the ENCODE consortium^30^. However, the two types of regulatory elements were generated using significantly different analytical pipelines^30,31^. Currently, it is not clear whether the difference in tissue types or the methods used to map the two different types of regulatory elements is responsible for the differential association with AP hotspots that are shared by multiple samples.

Even more strikingly, sample-shared hotspots exhibited a very strong tendency to map to just one gene, *Sfi1*, which encodes the mouse homolog of yeast spindle assembly associated Sfi1 protein (Supplementary Fig. 7b). For example, 203/2705 or 7.5% of hotspots in the mouse genome were shared by at least 2 samples mapped to that particular locus (Supplementary Data 18). Interestingly, the *Sfi1* gene locus—both exons and introns—also showed a significant (67.8% of the 61,575 bp) overlap with sequences representing embryonic promoters (Supplementary Fig. 7b). We then calculated the sequence coverage of all mouse genes by embryonic promoter sequences and found that *Sfi1* was ranked 283 out of 18,826 genes (larger than 5 kb) or was within approximately the top 1.5 percentile of such mouse genes ranked by this coverage. Thus, the association of AP sites and embryonic promoters is consistent with the high enrichment of these sites in the *Sfi1* locus (also see “Discussion”).

**Fig. 6:**
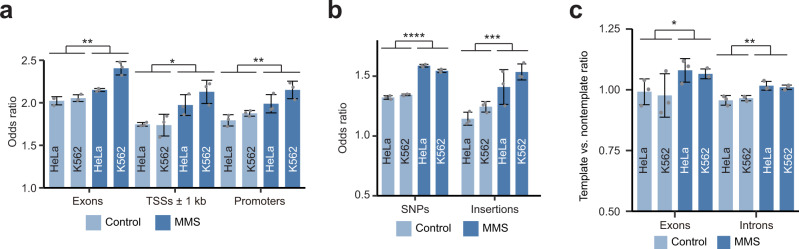
Properties of sample-level AP site hotspots in human cell lines treated with MMS. **a** Enrichment of hotspots in exons, introns and promoters in the samples treated with MMS (methyl methanesulfonate) and the corresponding controls in HeLa and K562 cell lines. **b** Enrichment of hotspots at SNPs and insertions in MMS-treated and control samples in HeLa and K562 cell lines. **c** Distribution of template vs. nontemplate ratios for hotspots in the exons and introns in the MMS-treated and control samples in the HeLa and K562 cell lines. **a**–**c** Hotspots were defined based on read depth ≥2. Asterisks above connecting lines indicate significance of difference between the MMS-treated and control samples as represented by raw *p*-values of ≤0.05 (*), ≤0.01 (**), ≤0.001 (***) and ≤0.0001 (****) calculated by the two-sided paired *t* test. Data are presented as mean values +/− SD based on three biological replicates. Source data are provided as a Source data file.

To further investigate the potential mode of hotspot generation, we identified hotspots in the K562 and HeLa cells that were treated for a short time (1 h) with MMS and in the corresponding control samples. MMS can mostly generate 7-methylguanine and 3-methyladenine, which are then converted into AP sites either via BER-mediated repair or directly by destabilizing the *N*-glycosyl bond^26^. However, since the half-lives of these adducts are 70 and 30 h, respectively, at 39 °C^38^, the AP sites produced by the short MMS treatment times should be mostly created by BER. Strikingly, compared to the hotspots found in the untreated controls, the hotspots found in the MMS-treated samples exhibited significantly higher enrichments in exons, in the immediate vicinity (±1 kb) of TSSs and in promoters^39^, as well as in sequence variants^40^ (SNPs and insertions) (Fig. 6a, b and Supplementary Data 5). Moreover, the AP site hotspots of the MMS-treated samples exhibited a significantly higher enrichment on the template strands of both exonic and intronic regions (Fig. 6c and Supplementary Data 5). These patterns were observed for both HeLa and K562 cell lines (Fig. 6a–c). The differences in the genomic distributions of hotspots between the treated and untreated samples should be due to the presence of hotspots that were specifically induced in response to MMS. Since those hotspots were most likely generated by BER (as opposed to spontaneous depurination), these results suggest that BER can generate hotspots of AP sites and that their distribution in the genome is not random (see “Discussion”).

### Nucleotide preference of AP sites

**Fig. 7:**
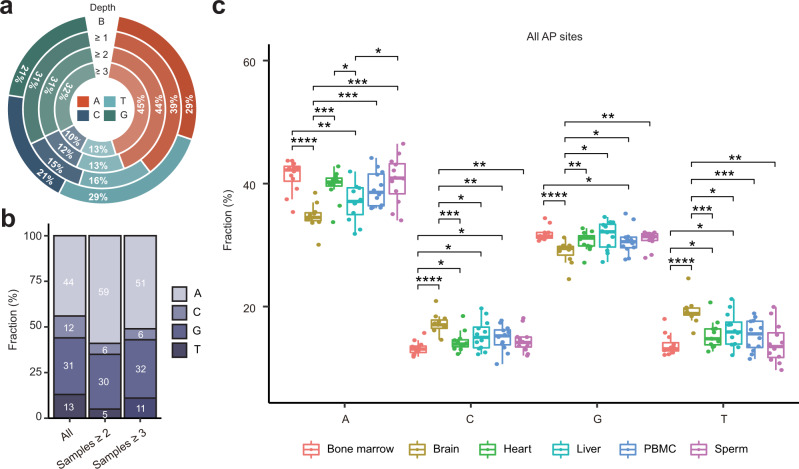
Nucleotide distribution of all AP sites in the mouse nuclear genome. **a** Average fractions of each nucleotide in the sequence of the nonrepeated portion of the mouse nuclear genome (“B”) as well as in all AP sites (“Depth ≥1”) or only hotspots (“Depth ≥2” or “Depth ≥3”) across the 71 samples. **b** Fraction of each nucleotide for all hotspots and those shared by the indicated number of samples. **c** Fraction of each nucleotide for all AP sites in different tissues (see Supplementary Fig. 8 for more information). Box plots indicate median (middle line), 25th, 75th percentile (box) and 1.5× interquartile range (whiskers) as well as each individual data (single points) based on 12 (3 biological replicates of 4 age groups) samples per tissue type with the exception of brain represented by 11 samples. Asterisks above connecting lines indicate the significance of differences between the indicated pairs of tissues as represented by raw *p*-values of ≤0.05 (*), ≤0.01 (**), ≤0.001 (***) and ≤0.0001 (****) calculated by the two-sided Wilcoxon rank-sum test. Source data are provided as a Source data file.

As shown in Fig. 7a, AP sites were significantly enriched in purines as follows: 70% of AP sites detected by ≥1 read mapped to adenines or guanines compared to the 50% expected by chance (*p*-value <2.2E−16, binomial test). Furthermore, the average fraction of AP sites mapping to adenines was significantly higher than that of guanines: 39% vs. 31% (*p*-value <2.2E−16, binomial test, Fig. 7a and Supplementary Data 19). The fraction of AP sites mapping to adenines increased in sample-level hotspots with increasing depth from 44% to 45%, while the fraction mapping to guanines reached 32% (Fig. 7a and Supplementary Data 19). Strikingly, the sample-shared hotspots showed an even stronger preference for adenines; for example, 59% of the hotspots shared by ≥2 samples were mapped to adenines compared to only the 44% found in at least one sample (*p*-value 2.2E−16, binomial test, Fig. 7b and Supplementary Data 20). On the other hand, the fraction of guanines remained fairly constant (30% vs. 31%) (Fig. 7b and Supplementary Data 20).

Interestingly, the tissue type also exhibited a significant effect on the nucleotide signature of the AP sites. The sites in the brain exhibited significantly lower enrichment in adenines and, to a lesser extent, guanines compared to other tissues while showing a significantly higher enrichment in thymines and, to a lesser extent, cytosines (Fig. 7c and Supplementary Data 19). For example, the median fraction of AP sites mapping to adenines in the brain was 34%, while in the bone marrow, it was 42% (Fig. 7c). The corresponding values for guanines were 29% vs. 31% (Fig. 7c). This effect could be seen at different depths of AP site detection (Supplementary Fig. 8 and Supplementary Data 19).

It is worth noting that SSiNGLe-AP has the following methodological caveat caused by the addition of a polyA tail (Fig. 1), which could affect the precision of AP site detection at or adjacent to adenines. In the context of 5’-BAN-3’, in which B is C, G or T, if an AP site occurs at either the A or N base, always maps to the A base. This could increase the fraction of AP sites mapping to the A bases. Nonetheless, the results above suggest that AP sites occurring at As or bases immediately following As have the following properties: (1) they are more likely to be found in the same samples and even more so, in different samples; and (2) their occurrence is more affected by the different biological conditions (e.g., tissue type) compared to AP sites mapping to other bases.

### Age-related changes in the genomic landscape of AP sites

To determine possible age-related changes in genomic profiles of AP sites, we calculated Spearman correlations between the various genomic features described above and age in each tissue. Interestingly, we could not find any feature that showed a consistent correlation with age in all 6 tissues. However, we found 4 prominent age-related signatures that were either common to most tissues or had specific patterns, as described below.

**Fig. 8:**
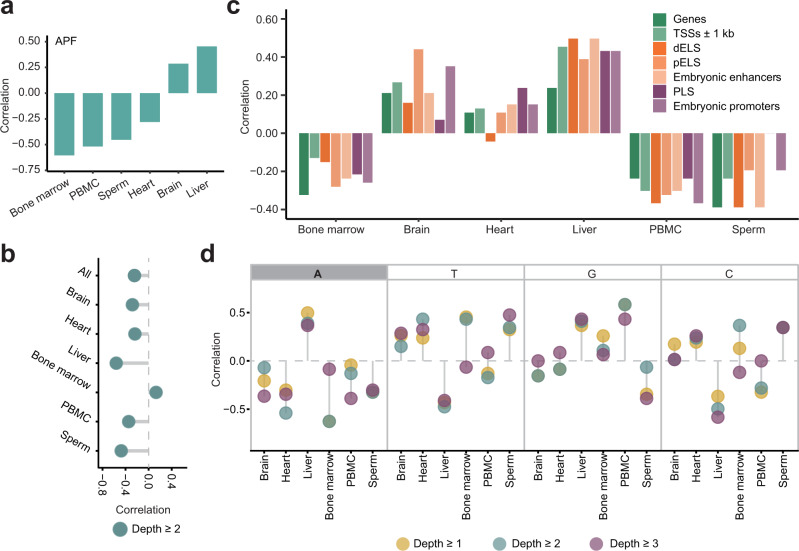
Association of various genomic features of AP sites and age. **a** Spearman correlation (*Y*-axis) between global levels of AP sites (APF) and age in different tissues (*X*-axis). **b** Spearman correlation (*X*-axis) between age and fractions of sample-level hotspots with depth ≥2 across all tissues or in the individual tissues (*Y*-axis). **c** Spearman correlation (*Y*-axis) between age and the odds ratios of association of AP sites with different genomic elements in different tissues (*X*-axis). The dELS, pELS and PLS represent candidate cis-regulatory elements with distal enhancer-like, proximal enhancer-like and promoter signatures, respectively. **d** Spearman correlation (*Y*-axis) between age and nucleotide distribution of AP sites detected with the indicated read depths in different tissues (*X*-axis). The most consistent negative correlation pattern between age and fraction of AP sites at A nucleotides is highlighted. Source data are provided as a Source data file.

First, as seen in Fig. 8a, 4 out of 6 tissues showed a negative correlation of APF with age ranging from −0.60 to −0.28, and only the brain and liver correlated positively with age (Supplementary Data 21). These results suggest that with the exception of those two tissues, there is a general negative correlation between the global number of AP sites and age.

Second, we found a tendency of AP hotspots to decrease with age in 5 out 6 tissues, with the exception of bone marrow (Fig. 8b). The correlation of the fraction of hotspots with a depth ≥2 and age among all samples was −0.25; however, the correlation reached −0.56 in the liver and −0.48 in sperm (Fig. 8b and Supplementary Data 21).

Third, for each tissue, we found an interesting tendency in which the occurrence of AP sites had a propensity to either favor genes and all types of regulatory elements with age or avoid them entirely (Fig. 8c, Supplementary Data 21). For example, Spearman correlations between age and odds ratios of overlap between AP sites and genes and every type of regulatory element were either all (or mostly) positive in brain, heart and liver or all negative in bone marrow, PBMC and sperm (Fig. 8c and Supplementary Data 21).

Fourth, we found that with age, the AP sites had a tendency to occur at bases other than adenines in all tissues except the liver (Fig. 8d and Supplementary Data 21). In other words, Spearman correlations between the fraction of AP sites mapping to A were negative in 5 out of 6 tissues for AP sites found with any depth (Fig. 8d and Supplementary Data 21).

### Distribution of AP sites in the mitochondrial genome

**Fig. 9:**
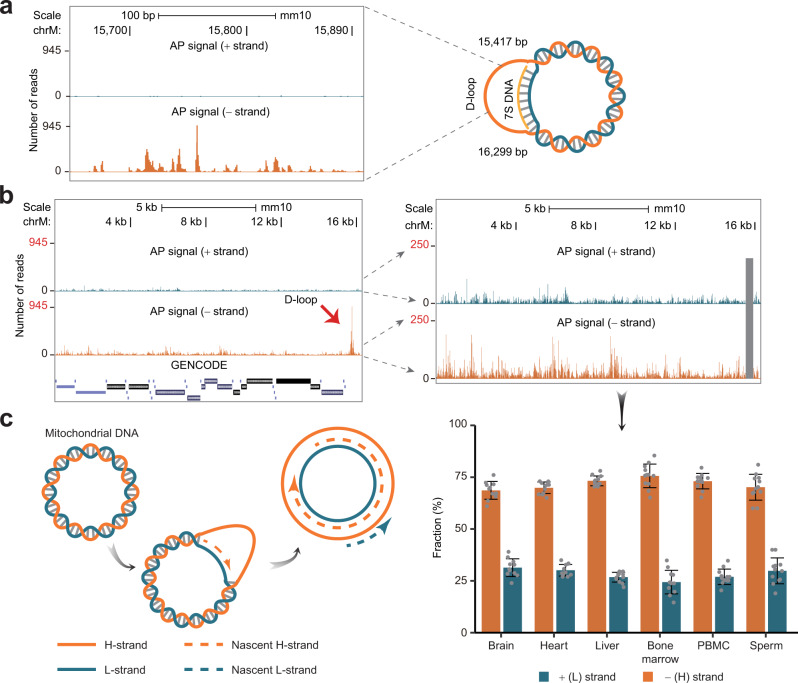
Distribution of AP sites in the mouse mitochondrial genome. **a** Distribution of AP sites in the D-loop region of the mouse mitochondrial genome. **b** Whole-chromosome view of the distribution of AP sites in the mitochondrial genome to show the dominance of the AP sites derived from the D-loop region (left). The view on the right represents the view with adjusted *Y*-axis and the D-loop region (with coordinated chrM: 15,650–15,900 bp) blocked by a gray box to show that the AP sites were also enriched on the minus (heavy) relative to the plus (light) strand of the genome outside of the D-loop region. The enrichment was further quantified in the histogram showing the fraction of AP sites mapping to the “+” or “−” strand in each tissue outside of the D-loop region. Data are presented as mean values +/− SD based on 12 (3 biological replicates of 4 age groups) samples per tissue type with the exception of brain represented by 11 samples. **c** Diagram of replication for the H (heavy) and L (light) strands of the mitochondrial genome (corresponding to the “−” and “+” strands, respectively) based on the commonly accepted strand displacement model^42^. The distribution of AP sites (*Y*-axes) is represented by the total number of reads corresponding to AP sites found at each nucleotide position across all 71 samples (**a**, **b**). Source data are provided as a Source data file.

The two strands of the mitochondrial genome differ in purine content, which is responsible for the differences in their buoyant densities. Given the association of AP sites with purines, we examined the distribution of AP sites in mitochondrial DNA. Strikingly, we noticed a localized region containing multiple very strong hotspots shared by multiple samples on the “−” or H (heavy) strand enriched in purines (Fig. 9a). Interestingly, this region localized to the D-loop region—a region involved in the formation of a specialized three-stranded structure formed during replication of mitochondrial DNA^41^.

However, even after removing the signal of AP sites from the region encompassing the hotspots (chrM:15,650–15,900) from both strands, we noticed a significant (2.55-fold) preference of AP sites toward the entirety of the H strand (Fig. 9b and Supplementary Data 22). However, the magnitude of the differences in the fraction of purines (53.11% for the H vs. 46.89% for the light (L) strand) could not explain the observed magnitude of difference observed in the AP sites. An alternative explanation could be provided by the mode of replication of the mitochondrial genome, in which the H strand is single-stranded for a period of time^42^ (Fig. 9c). Therefore, it is conceivable that the AP sites produced on the single-stranded H strand are either less efficiently repaired by APE1, which has lower activity on AP sites in single-stranded DNA^23^; caused by higher rates of spontaneous abasic site formation in single-stranded DNA^11,12^ or are protected from APE1 by the single-strand DNA binding protein coating the H strand^42^. While currently the reason behind this difference is not clear, the explanation based on the replication mode also highlights the fact that the various aspects of the nonrandom AP site profiles may not only be explained solely by the differences in kinetics of appearance of this DNA lesion but also in its repair (see more in “Discussion”).

## Discussion

Here, we describe a method, SSiNGLe-AP, to detect AP sites genome-wide and with nucleotide-level resolution. We extensively validated the method to show that SSiNGLe-AP exhibits high technical reproducibility, low background from unblocked breaks and AP sites caused by sample manipulation, and very high precision. The use of an enzyme to detect AP sites rather than complex organic chemistry makes this method compatible with the skillsets available in most molecular biological laboratories. Furthermore, the SSiNGLe-AP method can also be easily adapted to map other types of DNA damage by using different DNA repair enzymes, which recognize those lesions and convert them into SSBs. For example, this method can be extended to mapping uracil by incorporating UDG treatment. In another example, SSiNGLe-AP can be adapted to mapping oxidized purines such as 8-oxo-7,8-dihydroguanine (8-oxoG) by incorporating Fpg (Formamidopyrimidine DNA Glycosylase) treatment that converts this lesion into SSBs with 3’-phosphate termini^43^, followed by a phosphatase treatment to convert the termini into 3’OH that are mappable by SSiNGLe-AP.

It is worth noting that the APE1 enzyme used by SSiNGLe-AP to detect AP sites has multiple functions and activities. In addition to having very strong AP endonuclease activity, APE1 can also recognize various types of oxidation-caused damage to the 3’ ends, such as 3’ phosphates or 3’ phosphoglycolates, and process them to generate 3’OH termini^44^. However, DNA strands containing such oxidated 3’ ends are not detectable in the SSiNGLe-AP assay since they cannot be biotinylated by TdT, leading to a loss in these molecules at the bead capture step before APE1 cleavage (Fig. 1). On the other hand, APE1 can also recognize specific types of base damage, such as 5,6‐dihydro‐2′‐deoxyuridine and 5,6‐dihydrothymidine, and incise at these locations to generate 3’OH termini^45,46^ as part of the nucleotide incision repair (NIR) pathway^47^. SSiNGLe-AP can detect such types of DNA damage; however, they usually occur in response to specific types of DNA damaging treatments, such as radiation or treatment with certain carcinogens^45,46^. Therefore, the results of SSiNGLe-AP under conditions in which a significant background of NIR substrates is expected should be interpreted cautiously and potentially should be augmented by performing SSiNGLe-AP on MX-treated DNA, which should reduce APE1 cleavage at true AP sites.

In this work, AP site profiling in multiple sample types using SSiNGLe-AP has shown that the distribution of AP sites in the genome is not random but rather exhibits striking patterns of associations with different genomic features, which could be influenced by tissue type, age and other factors (e.g., transcription). Some of these patterns are consistent with the existing knowledge of AP site biology and, in some cases, could also augment the knowledge. One such example is the strong enrichment of the AP sites detected by SSiNGLe-AP in purine nucleotides, which was also identified by snAP-seq^19^ and is concordant with the well-known knowledge that purines are much more susceptible to spontaneous AP site formation^6^. The second example is the association of AP sites and transcription. Transcribed status is an important determinant of DNA repair efficiency^32,33^. Interestingly, we found that AP sites exhibited a higher tendency to occur on both template and nontemplate strands in more actively transcribed mouse genes, which was consistent with a previous report in which the accumulation of AP sites was found to increase significantly in highly transcribed yeast genes^48^. On the other hand, the stronger enrichment in AP sites on the nontemplate strands of the expressed mouse genes found in this study is consistent with the previous observation that AP sites are potent blockers of transcription in vitro^49^ and that yeast AP sites are preferentially removed from the transcribed strand via transcription-coupled nucleotide excision repair (NER)^50^. Therefore, our results extend previous in vitro and yeast studies to suggest that transcription status can have a complex effect on mammalian AP sites, affecting both the rates of their generation and repair. In the third example, which was consistent with the well-known high mutagenic potential of AP sites^6^, we found a significant association of AP sites with the sequence variants found in the normal population and with mutations that occurred in cancer cells. We found that the AP sites were enriched in insertions and SNPs but not deletions in both species and in multiple tissue and cell types. While SNPs have been previously associated with AP sites, at least in part via error-prone TLS^6^, to our knowledge, insertions have not been associated and therefore could represent an unidentified mutational signature of mammalian TLS. These results suggest that at least some sequence variants that occur normally could be explained by the AP sites that are likely found at certain nucleotides in the genome and lead to mutations.

One striking outcome of this work is the discovery that AP sites tend to occur more often at specific single-nucleotide positions—AP site hotspots—in mammalian genomes. This observation contradicts the results obtained with snAP-seq^19^. Currently, the reasons for the discrepancies between the studies are unknown, but it is quite possible that technical differences between the methods are responsible. Nonetheless, it is important to note that the genomic landscapes of AP sites as well as the existence and the genomic coordinates of the single-nucleotide hotspots, which were detected by SSiNGLe-AP using APE1, were also confirmed using nonhomologous AP endonuclease Endo IV and thus unlikely represent biases of APE1 toward certain sequences or affected by sequencing scale. In addition, while detectable, the bias of the SSiNGLe-AP procedure itself is relatively small. Altogether, these observations provide evidence against the technical bias underlying the existence of the single-nucleotide AP site hotspots detected by SSiNGLe-AP.

The existence of hotspots and the nonrandom distribution of AP sites in the genome in general is consistent with multiple previous studies showing that rates of DNA damage and DNA repair efficiency can have strong intragenomic variation. For example, rates of DNA damage and repair have been shown to depend on (1) chromatin state with active chromatin being more accessible to attack by DNA damaging agents^51^; (2) presence of single-stranded DNA that is more prone to abasic site formation^11,12^ and could exist, for example, in highly transcribed genes or replicating mitochondrial genome; (3) nature of functional genomic elements and local sequence contexts^52,53^. The intragenomic heterogeneity has also been shown for BER^54,55^. The profiling of AP site hotspots in response to the MMS treatment performed in this study is also consistent with these observations. Taken together, these results suggest that some hotspots found in normal tissues could also be generated by BER, potentially in response to endogenous DNA damage. However, currently, it is not possible to distinguish the relative genome-wide fraction of hotspots caused by BER vs. those caused by spontaneous depurination, considering that in mammalian cells, AP sites can be generated by 11 DNA glycosylases that are encoded by 10 genes with overlapping substrate specificities^8^.

Nevertheless, the observed patterns of enrichment in AP sites and hotspots may not simply reflect the mere byproducts of differential repair activity but are rather part of an emerging theme that connects DNA damage to the regulation of gene expression. For example, multiple reports have linked the DSBs formed at promoters with the activation of transcription^56–61^. Furthermore, AP sites in the promoters of multiple genes have also been directly linked to transcriptional control^62–64^. Therefore, the strong enrichment of the sample-shared AP site hotspots in the promoters and enhancers found in this work could signify a far wider involvement of AP sites in the regulation of gene expression than that previously anticipated. In this respect, *Sfi1* is expressed at a low level (median TPM of 0.74), suggesting that transcription is not a likely contributing factor to the wealth of the AP site hotspots found at that gene locus. On the other hand, the *Sfi1* gene has multiple promoter elements located within its boundaries that also have a strong association with sample-shared AP site hotspots. Since promoters can often regulate multiple genes^65^, similar to enhancers, it is conceivable that at least some of the hotspots located in that locus could participate in regulating the expression of genes other than *Sfi1*. However, it is not clear whether differential BER activity or other processes, such as increased local rates of DNA damage, are responsible for the abundance of AP site hotspots at this gene locus.

Another striking outcome of this work is the observation that tissue type can have a significant effect on many genomic patterns of AP sites. Since the procedure was the same for all the samples, these differences should reflect biological, rather than technical, differences in the distribution of AP sites in different tissue types. Most notably, the brain, followed by the liver, exhibited the most striking differences from other types of tissues. The brain had the lowest abundance of AP sites and the highest abundance of SSBs in all 6 tissues. Furthermore, in addition to the abundance, the patterns of AP sites in the brain exhibited striking differences from all tissues. First, when considering all AP sites mapping to the repeat and nonrepeat portions of the genome, the AP sites in the brain exhibited a striking enrichment in several classes of repeats, most notably satellite repeats, compared to that of all other tissues. However, even after considering only nonrepeat AP sites, the profiles in the brain were quite different, which was exemplified by the lowest enrichment of AP sites in exons and other functional elements. On the other hand, AP sites in the brain (and liver) have a tendency to occur more often on the template strand of exons and introns compared to those from other tissues.

However, the reasons for the differences in the profiles of AP sites found in the brain are unknown. Notably, the brain and liver had lower abundances of AP sites, higher abundances of SSBs and higher expression of *Apex1* mRNA than those of the other tissues. While the *Apex1* mRNA differences were not statistically significant, combined with the relative abundances of AP sites and SSBs, they might suggest that differences in BER activity could explain the differences in the abundances of these two lesions. However, the overall differences in BER efficiency cannot explain the differences in the enrichment of AP sites at specific genomic locations. Therefore, we hypothesize that, in addition to the global levels of BER activity, tissue-specific differences in BER activity at specific genomic locations or elements are likely responsible for the differences in genomic patterns among the different tissues. In this case, the genomic patterns of BER activity would be most different in the brain compared to other tissues. In fact, localized enrichment of SSB repair activity at specific genomic elements—enhancers—has recently been found in neurons^66^. Overall, the uniqueness of genomic patterns of AP sites found in the brain is interesting because of a number of neurological diseases connected to defects in various components of DNA repair^67^, including defects in the repair of SSBs^68^, the downstream intermediates of AP site repair. In this respect, our results suggest that in addition to the global load of DNA damage, genomic patterns of DNA damage should be considered since increased DNA lesions at certain genomic locations, for example, the template strand of exons or genes in general, could be a strong contributing factor to a disease state.

We found that certain features of genomic AP site profiles do change with age; however, tissue-type dependency was also a major influencing factor since we could not identify consistent changes that were common to all tissues. Unexpectedly, we found a decrease in the global number of AP sites with age in 4 out of 6 tissues, and the brain and liver were the only tissues that exhibited an age-dependent increase in AP sites. Ostensibly, these results contradict the abovementioned cornerstone importance of DNA damage in major theories of aging^4^. However, significant questions about the connection between DNA repair/damage and age remain^69^. For example, when results from 36 studies that measured global change in DNA damage in human subjects with age using different technologies were compiled in a meta-analysis^70^, only a relatively weak (albeit significant) positive correlation of 0.23 between DNA damage and age was revealed. That study also found a significant influence of tissue type, lifestyle and techniques used to measure DNA damage on the relationship between age and DNA damage^70^. Consistent with this work, our results suggest that the relationship between DNA damage and age is likely to be much more complex and nuanced than previously expected and have a strong dependency on the tissue type. Additional studies are needed to understand this relationship and the biological significance of all the findings reported here. Nevertheless, our results are consistent with the growing notion that the effects of DNA lesions are complex and nuanced and may have regulatory functions in addition to being unwanted byproducts of assaults on DNA. Overall, it appears that we are still at the beginning of understanding the relationships between the complexities of DNA damage and biological processes, including aging, and successfully exploring these complexities requires the development of multiple high-throughput methods that can map various types of DNA lesions with high precision and specificity genome-wide.

## Methods

### Ethical statement

The mouse experiments were approved by the laboratory animal management ethical review board of the School of Medicine, Huaqiao University (the protocol number A2021005).

### Biological material

Twelve male mice of the C57BL/6 J strain from 4 age groups (3, 12, 19, and 22 months old) were used to extract brains, hearts, livers, bone marrow, PBMCs and sperm. All mice were housed in specific pathogen-free conditions with 12-hour light/dark cycle, ambient temperature at 22–25 °C, 40–60% humidity, and access to food and water ad libitum at Xiamen University Laboratory Animal Center. Red blood cells in the bone marrow were removed with Red Blood Cell Lysis Buffer (Beyotime, C3702-500ml). PBMCs were isolated from blood using Ficoll-Paque PLUS (GE Healthcare, 17-1440-03). The motile spermatozoa were isolated from the mouse epididymal tail by collecting cells floating in the upper layer of RPMI 1640 (Thermo Fisher Scientific, C11875500BT). The isolated tissues were immediately placed into liquid nitrogen to quickly freeze. The whole organs were cut into small pieces and ground into powder by a tissue homogenizer in the presence of liquid nitrogen to avoid heating. All ground tissue samples were stored at −80 °C prior to DNA isolation. The mouse experiments were performed by Amogene (Xiamen).

The human CML leukemia cell line K562 (TCHu191) was obtained from the Cell Bank of the Chinese Academy of Sciences, and the human cervical carcinoma HeLa cell line (1101HUM-PUMC000011) was obtained from the National Infrastructure of Cell Line Resource. Cells were maintained in RPMI 1640 (Thermo Fisher Scientific, C22400500BT) supplemented with 10% (v/v) fetal bovine serum (ExCell Biotech, FSP500) and 1% (v/v) penicillin–streptomycin solution (HyClone, SV30010) at 37 °C in 5% CO_2_. Two million K562 or HeLa cells were seeded at 1 million cells per ml of culture medium without penicillin–streptomycin solution in 6-well plates. After 16 h, cells were treated with 1.5 mM MMS (Sigma, 129925) for 1 h or grown without MMS as a control.

### DNA isolation

Genomic DNA from the human cell lines and murine brain, bone marrow, heart, liver and PBMCs were isolated using a TIANamp Genomic DNA kit (Tiangen, DP304-03) with RNaseA (Tiangen, RT405-12) treatment according to the manufacturer’s protocols without the optional step of incubation with proteinase K at 56 °C. Genomic DNA from sperm cells was extracted using a Sperm DNA Purification Kit (Simgen, 4202050) following the manufacturer’s instructions. All DNA samples were eluted in UltraPure™ DNase/RNase-Free Distilled Water (Invitrogen), and the concentration was measured using SMA6000 (Merinton) and stored at −20 °C.

### Spike-in DNA preparation

The spike-ins were represented by three nonoverlapping double-stranded DNA fragments (500 bp) that were amplified from the LwCas13a gene cloned into the pC034-LwCas13a-msfGFP-2A-Blast plasmid, a gift from Dr. Feng Zhang (Addgene plasmid #91924; http://n2t.net/addgene:91924; RRID: Addgene_91924). The plasmid DNA (10 pg) was mixed with 20 µl of PCR solution (1× Taq buffer, 1 U Taq DNA polymerase (Tiangen, ET101), 0.5 µM of each forward (FP) and reverse (RP) primers, 0.2 mM dNTP mix (Takara, 4030) and 1.6 nM dUTP (New England BioLabs, N0459S). The PCR conditions were as follows: initial denaturation at 94 °C for 3 min; 30 cycles of denaturation at 94 °C for 30 s, annealing at 55 °C for 30 s and extension at 72 °C for 1 min; and further extension at 72 °C for another 7 min. After amplification, half of the PCR product was purified by 2× volumes of VAHTS DNA Clean Beads (Vazyme, N411-01) and served as the negative spike control without AP sites. The remaining 10 µl of the reaction was directly treated with 5 U UDG (New England BioLabs, M0280S) at 37 °C for 2 h to generate AP sites and to serve as the positive spike control, followed by purification with 2× volumes of VAHTS DNA Clean Beads. The concentrations of the spike-in DNAs were measured by SMA6000 (Merinton). The fragment size of each spike-in DNA was checked on a 1% agarose gel. The PCR primer pairs used to amplify the spike-ins S1, S2 and S3 were S1-FP: CCTGATCGAGAACAGCAAGAAGC; S1-RP: TCAGGAAGGCCTCGTTCTGCC; S2-FP: CCGGCCACTACAAGTATCAGAAG; S2-RP: CAACTCGTCGCTGAAGGTTTCTT; S3-FP: GGACTTCGAGCTGGAAGCCAAC; S3-RP: GCCGGAATCTCAGGTCCCG.

### SSiNGLe-AP

#### DNA fragmentation

Genomic DNA (1 µg) was incubated with 0.025 U TURBO™ DNase (Invitrogen, AM2239) in a total volume of 20 µl at 37 °C for 3 min followed by an additional incubation at 75 °C for 10 min in the presence of 15 mM EDTA (Invitrogen, 15575020). DNA input with low concentration was concentrated by Concentrator plus (Eppendorf). The digested DNA was purified by 2× volumes of VAHTS DNA Clean Beads and eluted in 15 µl of H_2_O supplemented with 750 pg of spike-in DNA mix containing equal mass of DNA from each spike. An aliquot (1%) of the eluted genomic DNA with spike-ins was used to generate a sequencing library directly to serve as the unblocked control.

#### Preparation of MX-treated DNA

HeLa genomic DNA (1 µg) was fragmented by DNase and purified by 2× volumes of VAHTS DNA Clean Beads. The DNA was incubated at 37 °C for 30 min in 20 µl of 50 mM K_3_PO_4_ (pH 7.0) buffer with or without 33 mM MX (Abmole, M10541). The DNA was recovered by ethanol precipitation, dissolved in 15 µl water and subjected to the SSiNGLe-AP procedure starting from the blocking step preceded by denaturation.

#### Free 3′OH end blocking

The eluted DNA from above was mixed with 2 µl of 10× Terminal Transferase Reaction Buffer and 2 µl of 2.5 mM CoCl_2_ and denatured at 95 °C for 30 s followed by rapid snap-cooling on ice, followed by the addition of 1 nmol biotin-11-ddCTP (Jena Bioscience, NU-850-BIOX-L) and 20 U TdT (New England BioLabs, M0282) and incubation at 37 °C for 4 h. The reaction was denatured again at 95 °C for 30 s, and 20 U TdT was added and incubated at 37 °C for another 4 h, followed by 10 min at 70 °C. The DNA was purified by 2× volumes of VAHTS DNA Clean Beads and eluted in 20 µl H_2_O.

#### Enrichment

BeaverBeads™ Streptavidin (BEAVER, 22308-1) were washed with Buffer I (10 mM Tris-HCl (pH 7.5), 1 mM EDTA, 1 M NaCl, 0.1% Tween-20) twice following the manufacturer’s instructions. Each eluted sample from above was mixed with 20 µl prewashed streptavidin-coated beads and Buffer I to a final volume of 100 µl followed by incubation at room temperature for 2 h with rotation to enrich the biotinylated blocked ssDNA. The beads were collected by magnetic stand and washed five times with 500 µl of Buffer I and once with 500 µl of 1× NEBuffer 4 (50 mM potassium acetate, 20 mM Tris-acetate, 10 mM magnesium acetate, 1 mM DTT).

#### APE1 treatment

The AP sites were cleaved by resuspending the bead-DNA complexes in 20 µl reaction solution containing 1× NEBuffer 4 and 10 U APE1 (New England BioLabs, M0282) with incubation at 37 °C for 2 h. Incubation without APE1 enzyme was performed to serve as an untreated control. After collecting the beads with a magnetic stand, the supernatant was collected and purified with 2× volumes of VAHTS DNA Clean Beads and eluted in 15 µl H_2_O.

#### Endo IV treatment

Alternatively, after capture, the beads were washed five times with 500 µl of Buffer I, once with 500 µl of 1× NEBuffer 3 (100 mM NaCl, 50 mM Tris-HCl, 10 mM MgCl_2_, 1 mM DTT) and resuspended in a 20 µl reaction system including 20 U Endonuclease IV (New England BioLabs, M0304S) and 1× NEBuffer 3 followed by 2 h incubation at 37 °C.

#### Library construction and sequencing

The purified DNA released from the beads was used as input at the polyA-tailing step of the SSiNGLe-ILM protocol^71^ in which no denaturation step was performed before the tailing step. Specifically, the purified DNA was incubated at 37 °C for 30 min in 22 µl solution containing 2 µl of 10× TdT buffer, 2 µl of 2.5 mM CoCl_2_, 4 U TdT and 20 nmol dATP (Takara, 4026), followed by addition of 20 nmol ddNTP (Roche, 03732738001) and continuing the incubation at 37 °C for 30 min, and inactivation at 70 °C for 10 min. After purification with 2× volumes of VAHTS DNA Clean Beads, the DNA was amplified by linear PCR with polyT primer (oligo-d(T)50-r(T)3 oligo). The linear PCR cycling conditions were as follows: initial denaturation at 94 °C for 30 s; 10 cycles of denaturation at 94 °C for 1 min, annealing at 55 °C for 30 s and extension at 72 °C for 30 s. The PCR product was purified with 2× volumes of VAHTS DNA Clean Beads, denatured at 95 °C for 5 min and tailed with dCTP (Takara, 4028) using the same procedure used for the polyA-tailing. The tailing reaction was purified with 2× volumes of VAHTS DNA Clean Beads and all purified DNA was PCR-amplified with P5_N10G10 (CTACACGACGCTCTTCCGATCTAGTTGCGGATGGGGGGGGGGHN) and P7_T12 (GTTCAGACGTGTGCTCTTCCGATCTTTTTTTTTTTTTVN) primers. The first round PCR cycling conditions were as follows: initial denaturation at 94 °C for 3 min; further denaturation at 94 °C for 30 s, annealing at 55 °C for 1 min and extension at 72 °C for 1 min; denaturation at 94 °C for 30 s, incubation at 37 °C for 1 min and slow ramp at 2 °C per minute to 72 °C followed by 2 min incubation; 30 cycles of denaturation at 94 °C for 30 s, annealing at 60 °C for 30 s and extension at 72 °C for 30 s; and final extension at 72 °C for another 7 min. The PCR product was purified by 2× volumes of VAHTS DNA Clean Beads and subjected to the 2^nd^ round PCR with Illumina-P5 (AATGATACGGCGACCACCGAGATCTACACTCTTTCCCTACACGACGCTCTTCCGATCT) and Illumina-P7 (CAAGCAGAAGACGGCATACGAGAT(index)GTGACTGGAGTTCAGACGTGTGCTCTTCCGATCT) primers. The second round PCR cycling conditions were as follows: initial denaturation at 94 °C for 3 min; 6 cycles of denaturation at 94 °C for 30 s, annealing at 55 °C for 30 s and extension at 72 °C for 30 s; and further extension at 72 °C for another 7 min. The library was purified by 2× volumes of VAHTS DNA Clean Beads. All PCR amplifications were performed in 20 µl solution containing 1× Taq buffer, 1 U Taq DNA polymerase, 0.2 mM dNTP mix, and 0.5 µM of each indicated primer. Sequencing was performed on the Illumina NovaSeq 6000 platform using a paired-end 150 bp (PE150) strategy by Novogene Corporation (Beijing), and 2 GB of raw sequence data were collected per library, with the exception of the K562 cells containing either positive or negative spike-in samples (1 GB), MMS-treated and untreated samples (5 GB) and the 8 mouse deep-sequenced samples and the corresponding unblocked controls (20 GB).

### SSB profiling

Genomic DNA (100 ng) was used directly as input into the SSiNGLe-ILM protocol^71^ at the polyA-tailing step as previously described^72^. The procedures were same as those mentioned in the *Library construction and sequencing* with two exceptions that the polyA-tailing was preceded by denaturation at 95 °C for 5 min and 12 cycles were used in the second round of PCR. Sequencing was performed on the Illumina NovaSeq 6000 platform using the same strategy as for the SSiNGLe-AP libraries.

### RNA-seq

Total RNA from the mouse samples was extracted using TRNzol Universal (Tiangen, DP424) and Total RNA kit I (Omega, R6834-02) according to the manufacturer’s protocols. The RNA samples were used for RNA-seq library construction using rRNA depletion and a strand-specific strategy. The library construction and sequencing using the Illumina NovaSeq 6000 platform with the PE150 strategy on the 10-GB scale were outsourced to Novogene Corporation (Beijing).

### Bioinformatics analysis

#### AP site mapping

Raw or quality filtered NGS reads were used to map AP sites in spike-ins or genomic DNA, respectively. The paired-end sequences from fastq files were first filtered for the presence of AGTTGCGGATG9-11 at the beginning of read 1 and T11-13 at the beginning of read 2, and then the AGTTGCGGATG9-11 and T11-13 sequence tags were removed. The filtered and trimmed reads were then mapped separately to the sequences of spike-ins, nuclear or mitochondrial mouse genomes (GRCm38/mm10) and the human genome (GRCh37/hg19) by BWA (version 0.7.17-r1188). For spike-ins, the positions of reads 2 that achieved the following criteria were extracted: (1) Mquality ≥20, (2) reads 1 and 2 mapped uniquely to different strands with distance <500 bp, (3) the first base of read 2 was matched. For the nuclear and mitochondrial genomes, the positions of read 2 that met the following criteria were extracted: (1) PCR duplicates were removed using the Picard suite (version 2.0.1). (2) Mquality >20; (3) the “Flag” value of read 2 in SAM format was 147 or 163; (4) the first base of read 2 was matched. Fractions of thymines in 20 genomic bases upstream of the read 2 positions were calculated and the positions where the corresponding fractions exceeded 40% were removed using previously published code^20,71,73,74^. Then, the coordinate of one base upstream of the first aligning base of each remaining read 2 was assigned as the position of the AP site, with the strand of the site being the strand opposite of the strand of the read.

#### Spike-in analysis

The background of free 3’OH termini was defined as the fraction of reads representing sites mapping to the four 3’-most bases of the spike-in sequences relative to the reads that represented all sites mapping to the spike-ins. To estimate the maximum background of artificial AP sites based on spike controls, we prepared 3 pairs of libraries containing positive and negative spike-ins in parallel (Fig. 2a, e) and calculated the fraction of reads representing AP sites in each negative spike-in library compared to the number of such reads in the corresponding positive spike-in library (Supplementary Data 1). The median fraction of the 3 replicates was then used to estimate the background. The relative depth of sites for each nucleotide position within each spike-in sequence and within each sample was calculated as the number of reads of each site divided by the total number of reads of all sites in the 3 spike-ins in the sample (Fig. 2d). The hotspots used for the nucleotide distribution analysis in Fig. 2d were defined as single-nucleotide positions with relative depths higher than the threshold and higher than those of flanking nucleotides. To visualize the distribution of sites in the 3 pairs of libraries that contained positive and negative spike-ins (Fig. 2e), for each pair of libraries, we calculated the normalized relative depth by normalizing the read depth at each position by the total number of reads in each library and by the total fraction of reads mapping to the corresponding strand of the spikes-in in both libraries.

### Definition of different types of AP hotspots detected in the mouse genome

1. Sample-level hotspot: a single-nucleotide position in the genome detected with a depth of at least 2 reads in the same sample.
2. Sample-shared hotspot: a sample-level hotspot shared by multiple samples of the same or different tissues or ages.
3. Tissue- or age-specific hotspot: a single-nucleotide position in the genome that is (1) shared by at least 2 samples from the same tissue type or age group, respectively, with a depth ≥ 2 in each; and (2) detected with a depth of ≤ 1 in any sample from any other tissue type or age group.

#### General analysis

Simulation of AP sites was performed in R (version 4.1.0), and the random assignment of coordinates in the nonrepeat genomic space was based on BEDOPS^75^ (version 2.4.40). The latter was also used to identify sample-level hotspots. BEDTools (version 2.30.0) was used to identify other types of hotspots and calculate the overlaps between AP sites and different genomic features. The nucleotides corresponding to AP sites were extracted using the “getfasta” function of BEDTools. The hotspots in the spike-ins were identified in R (version 3.6.0). BEDTools (version 2.29.2) was used to extract the nucleotides corresponding to AP sites in the spike-ins. The R package “ggpubr” was used to calculate the *p*-values of the odds ratios of different features and of the template vs. nontemplate ratios using the two-sided binomial test. The *p*-values and 95% confidence intervals are provided in Supplementary Data 5–8, 11–13, 16, and 17. All tests based on the Wilcoxon rank-sum or the Wilcoxon signed rank test were also two-sided.

#### Estimation of the technical bias of the method

The Pearson correlations of the read depths at all AP sites (or hotspots at depth ≥2) found in the mouse genome (including the repeat regions) by the SSiNGLe-AP procedure with the read depths found at these sites in the “unblocked” controls were calculated, and then the coefficients of determination *r*^2^ were used to estimate the variances attributable to the steps common with the “unblocked” control.

#### Calculation of the abundances of AP sites and SSBs

The DNA size distribution profiles of the SSiNGLe-AP libraries were obtained using an Agilent 5400 Fragment Analyzer System with DNF-915 dsDNA reagent kits and used to estimate the APL metric—the fraction of DNA in the range of 180–1000 bp, which was larger than the estimated maximum primer dimer size of ~160 bp, relative to the total amount of DNA in the range of 1–1000 bp. The analysis was performed using the PROSize2.0 software and outsourced to Novogene Corporation (Beijing). The APF or ASF metric was calculated as the number of read pairs corresponding to AP sites or SSBs, respectively, divided by the number of read pairs starting with the expected polyT and polyG-containing sequences in the same sample.

### Calculations of the odds ratios of enrichment of all AP sites or hotspots for the different overlap analyses

1. Overlap of the AP sites or hotspots between pairs of different samples. For the technical reproducibility analysis, the expected fraction was calculated as the number of positions corresponding to AP sites or hotspots in replica 1 divided by the length of the genome. The observed fraction was calculated as the number of positions corresponding to AP sites or hotspots shared by both replicas divided by the total number of such positions in replica 2. To calculate the odds ratios for the APE1 vs. Endo IV comparisons, APE1 and Endo IV samples were treated as replicas 1 and 2, respectively. This analysis was based on the entire genome (unique and repeated sequences).
2. Enrichment of AP sites in the different types of repeats. The expected fraction was calculated as the total length of all repeated elements of a given type divided by the length of the genome. The observed fraction of all AP sites was calculated as the number of AP sites mapping to all repeated elements of that type divided by the total number of AP sites in the sample. In this analysis, AP sites with the same coordinates were counted separately.
3. Enrichment in the different types of genomic elements and sequence variants. The observed and expected fractions were calculated using the same strategy as described in #2, with the exception that the genomic bases corresponding to repeats were masked for all calculations and the coordinates of AP sites were collapsed such that AP sites with the same coordinates were counted only once.
4. For 1–3, the odds ratios of enrichment were calculated by dividing the observed fraction by the expected fraction.

#### Genome resequencing of HeLa cell line

Purified genomic DNA from our HeLa clone was used to perform library construction and sequencing on the Illumina NovaSeq platform using the PE150 strategy and 90-GB scale by Novogene Corporation (Beijing). Quality-filtered reads were aligned to the human genome by BWA (version 0.7.8-r455) and filtered for improper alignments by Samblaster (version 0.1.21). After removing duplicate reads by SAMtools (version 1.0) and Sambamba (version 0.4.7), the remaining alignments were used for variant calling using SAMtools. The above analysis was outsourced to Novogene Corporation (Beijing). Heterozygous C > G, C > A and C > T mutations in the TpCpN context were then selected. The expected fraction of each such mutation type was then calculated as the number of each type of such mutations in the HeLa genome divided by the total number of these three types of mutations. Then, for each sample, the corresponding observed fraction was calculated the same way, but for the mutation positions that overlapped with AP sites. Finally, the odds ratios of enrichment of AP sites at each type of heterozygous C > A/G/T mutation were calculated for each sample by dividing the observed fraction by the corresponding expected fraction.

#### RNA-seq analysis

Quality-filtered NGS reads were aligned to the genome using RSEM (version 1.2.28). The TPM values for annotated transcripts were estimated using the “rsem-calculate-expression” function of RSEM (version 1.2.28) with a strand-specific strategy. The TPM values of the longest transcript variants (based on the combined length of all exons) were selected to represent the expression levels of the corresponding genes. All known genes were classified into three categories—nonexpressed genes with TPM ≤ 1, low expressed genes with 1 <TPM ≤ 10 and highly expressed genes with TPM > 10. For each sample, each of the 3 types of genomic elements (exons, introns and regions ±1 kb around TSSs) associated with each annotated gene were stratified into 3 groups based on the expression of the corresponding gene and overlapped with the AP sites found in the same sample.

### Statistics and reproducibility

For assessing technical reproducibility, 2 or 3 technical replicates represented by independent NGS libraries from the same DNA sample were used. Since the reproducibility was very high, no additional replicates were generated. Reproducibility of SSiNGLe-AP detection of AP sites in the bacterial spike-ins in the background of human K562 DNA was performed using 3 technical replicates. Technical reproducibility of AP site detection by SSiNGLe-AP in mammalian genomes was assessed using 2 technical replicates of the 4 deep-sequenced mouse samples (8 samples total). Reproducibility of AP site detection with Endo IV enzyme was performed using 3 or 2 technical replicates for K562 or mouse samples, respectively. Most of the samples used to extract the patterns of AP sites in mammalian genomes were based on 3 biological replicates (represented by either different batches of cells or animals) for the same treatment or age/tissue combination that constitute the minimal accepted replicate number based on the established conventions and requirement for statistics in life sciences^76^. However, many comparisons done in this study were based on larger number of independent biological samples when samples of the same age or tissue were combined as indicated in the figure legends. Specifically, AP site detection in the MMS and MX experiments was performed using 3 biological replicates for each treatment or control. Profiling of AP sites, SSBs and transcriptome analysis using RNA-seq were generated using 3 different animals for each of the 24 combinations of the 6 tissue/cell types and 4 age groups, with the exception of a 22-month-old mouse brain sample that was excluded from the downstream analysis based on the blocking efficiency of spike-ins. With the exception of the spike-in sequences in the background of human K562 DNA, NGS reads or read pairs were excluded at the stage of the standard NGS QC filtering and PCR duplicate removal. Randomization and blinding were not applicable to this work since this study did not have treatment/control groups that were evaluated using subjective expert assessment. Furthermore, all animals had the same genetic background, gender and were raised under the same conditions.

### Reporting summary

Further information on research design is available in the Nature Research Reporting Summary linked to this article.

## Supplementary information

Supplementary Information

Description of additional Supplementary File

Dataset 1

Dataset 2

Dataset 3

Dataset 4

Dataset 5

Dataset 6

Dataset 7

Dataset 8

Dataset 9

Dataset 10

Dataset 11

Dataset 12

Dataset 13

Dataset 14

Dataset 15

Dataset 16

Dataset 17

Dataset 18

Dataset 19

Dataset 20

Dataset 21

Dataset 22

Reporting Summary

## Data Availability

The NGS data and the coordinates of AP sites generated in this study have been deposited in the GEO database under accession code GSE190955. The processed data generated in this study are provided in the Supplementary Data files and the Source data file and referred to in the main text, figure legends, and “Methods.” Overlaps between AP sites and various genomic elements were calculated using the datasets 1–4 below that were downloaded either from the UCSC Genome browser (datasets 1–4) using databases based on the mouse GRCm38/mm10 (http://hgdownload.soe.ucsc.edu/goldenPath/mm10/database/) or human GRCh37/hg19 (http://hgdownload.soe.ucsc.edu/goldenPath/hg19/database/) assemblies, or other sources (datasets 4), as specified below. (1) Annotated genes: GENCODE VM23 (mouse, file name: knownGene.txt.gz) and UCSC Genes (human, file name: knownGene.txt.gz). (2) Naturally occurring sequence variants: dbSNP 142 (mouse, file name: snp142.txt.gz) and dbSNP 151 (human, file name: snp151.txt.gz). (3) Repeats: RepeatMasker database (file name: rmsk.txt.gz for each species). (4) Regulatory elements: (a) Mouse Embryonic: The 15 chromatin states of embryonic mouse tissues were downloaded from http://renlab.sdsc.edu/renlab_website//download/encode3-mouse-histone-atac/ (file UCSC_ENCODE3_mouse_15_elements_pooled.zip); (b) Mouse cCREs: https://www.encodeproject.org (file UMass_ENCODE3_regulatory_regions.tar.gz); (c) Human chromatin state segmentation by HMM from ENCODE/Broad for nine human cell lines were downloaded from http://genome.ucsc.edu/cgi-bin/hgTrackUi?hgsid=1430833417_WK0z1Wy8QkJQmQyjoDR0Hv64atVQ&g=wgEncodeBroadHmm. For each element type (e.g., promoters), the coordinates of all subtypes of each element were merged from all cell lines to generate one set of coordinates used in the downstream analysis. Mouse and human genomic sequences were downloaded from http://hgdownload.cse.ucsc.edu/goldenPath/mm10/bigZips/chromFa.tar.gz and http://hgdownload.soe.ucsc.edu/goldenPath/hg19/bigZips/chromFa.tar.gz. Source data are provided with this paper.

## Source data

Source Data

## Code availability

Custom scripts constituting the pipeline used to generate genomic coordinates of AP sites from the sequencing reads were same as the previous scripts used for detection of SSBs by SSiNGLe-ILM^20^. These are available from GitHub^74^ and Zenodo^73^. The additional adjustments and downstream analyses involve standard packages in the R environment and BEDTools suite as described in “Methods.”
