# Supplementary material for: Complex genomic patterns of abasic sites in mammalian DNA revealed by a high-resolution SSiNGLe-AP method
Source: Nat Commun. 2022 Oct 5;13:5868. doi: 10.1038/s41467-022-33594-1 (PMC9534904; doi:10.1038/s41467-022-33594-1)
Supplement: Supplementary file 1 — Supplementary Information [file 41467_2022_33594_MOESM1_ESM.pdf]

Supplementary Information

Complex Genomic Patterns of Abasic Sites in Mammalian DNA Revealed by a High-Resolution SSiNGLe-AP Method

Cai et al.

Supplementary Figures

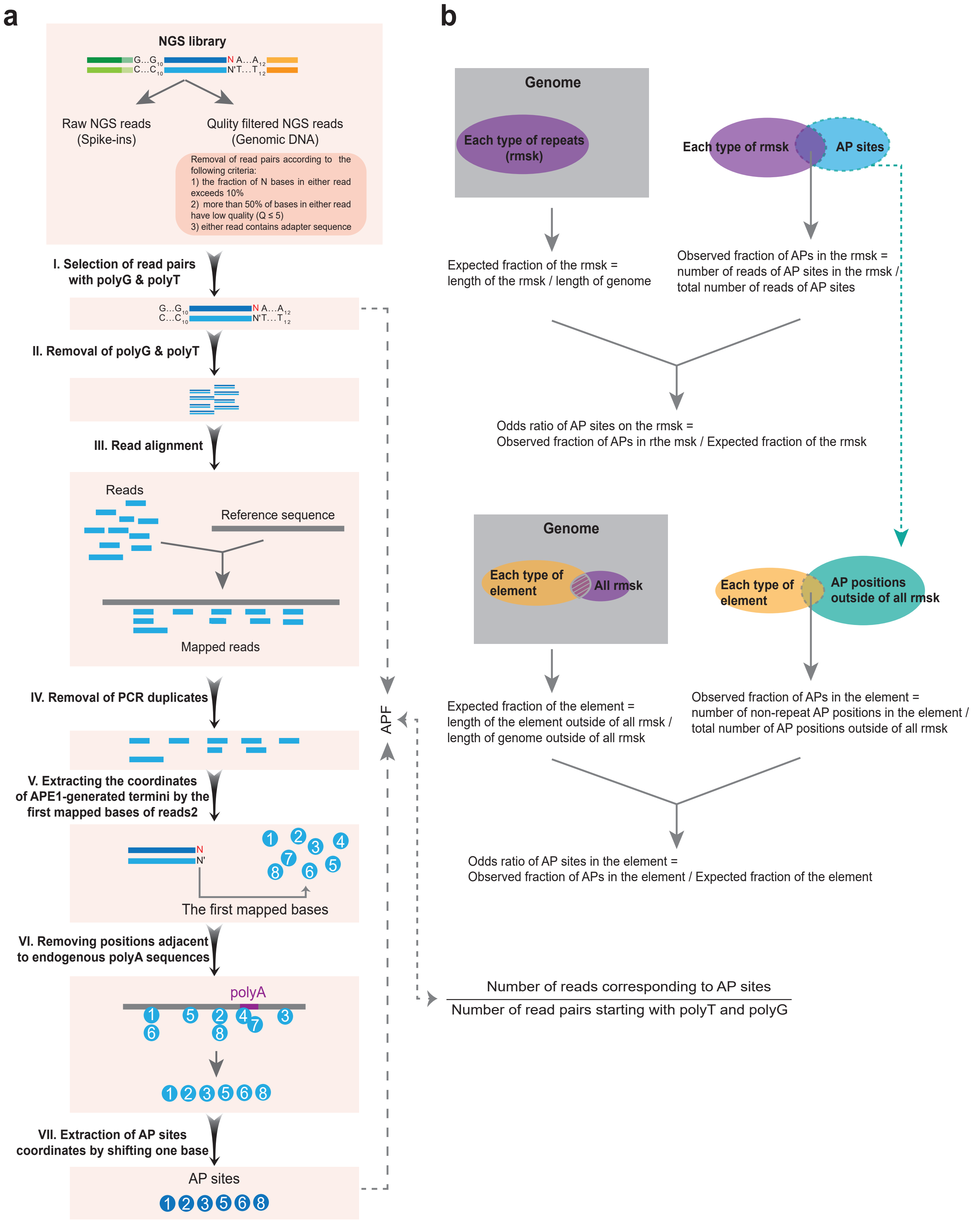

Supplementary Figure 1. Schematics of the analytic part of SSiNGLe-AP. a Workflow of extraction of AP sites starts with selecting read pairs that start with the polyG and polyT sequences of expected lengths on reads 1 and 2, respectively, and correspond to primers that hybridized to the polyC and polyA tails. The polyG and polyT sequences were then removed, followed by mapping of the trimmed reads to the reference sequence. The aligned read pairs were then subjected to PCR duplicate removal and those mapping uniquely in the genome were then selected for the downstream analyses. The coordinates of the first mapped bases of read 2 corresponding to the APE1-generated 3'OH termini were then extracted and further filtered out to remove those located adjacent to the endogenous polyA regions. The coordinates of the positions that survived this step are then shifted one base to represent the actual AP sites. Processing the reads derived from the spike-ins follows essentially the same pipeline, with the exception that no PCR duplicates are removed; given the short size of the corresponding DNA fragments, this step would remove all reads. **b** Illustration of the odds ratio calculations used to estimate the significance of overlap between AP sites and various classes of repeats and genomic elements.

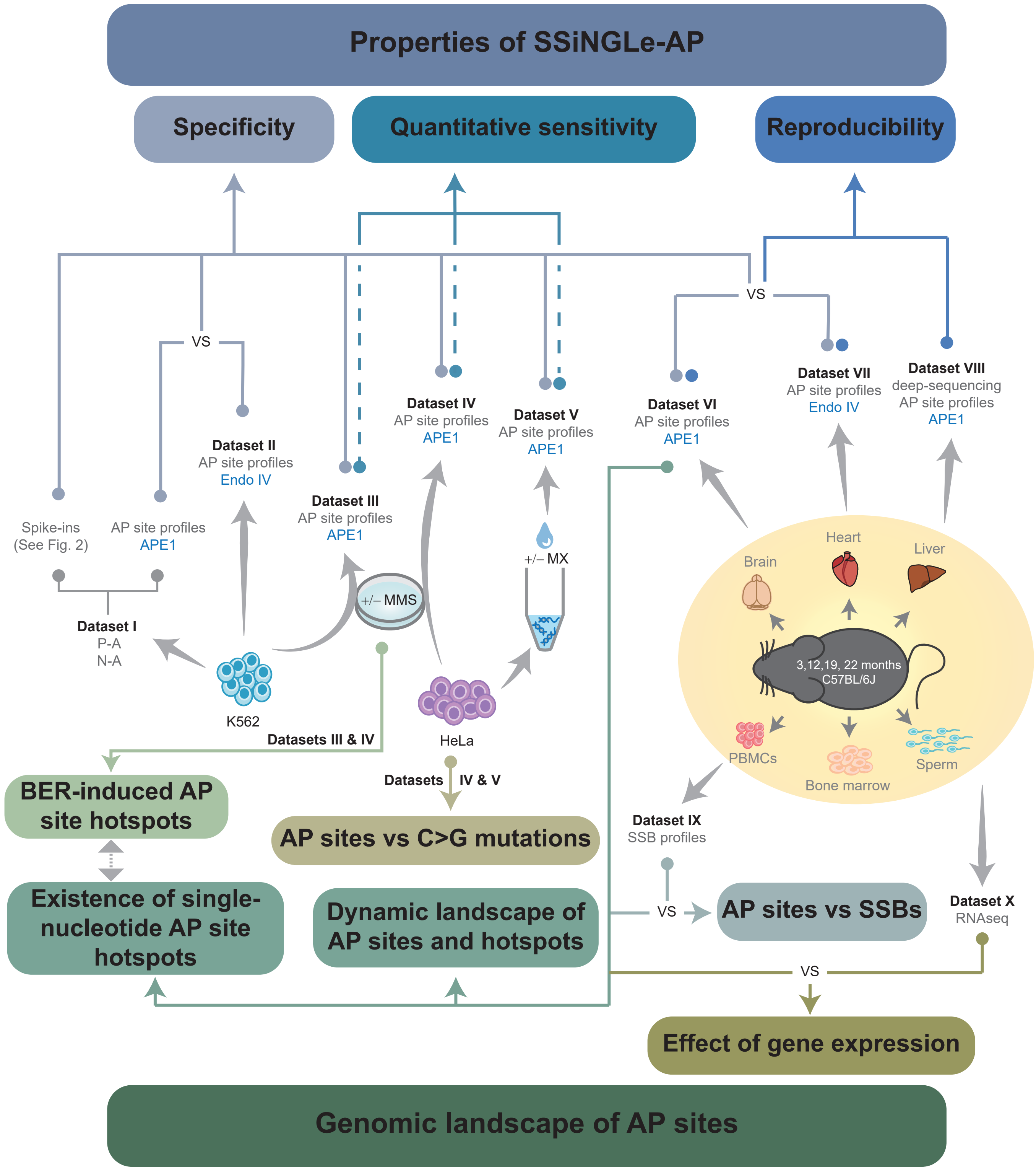

Supplementary Figure 2. Flow chart diagram illustrating the overall concept of the project. The relationships between the different datasets and major conclusions generated in this work are shown.

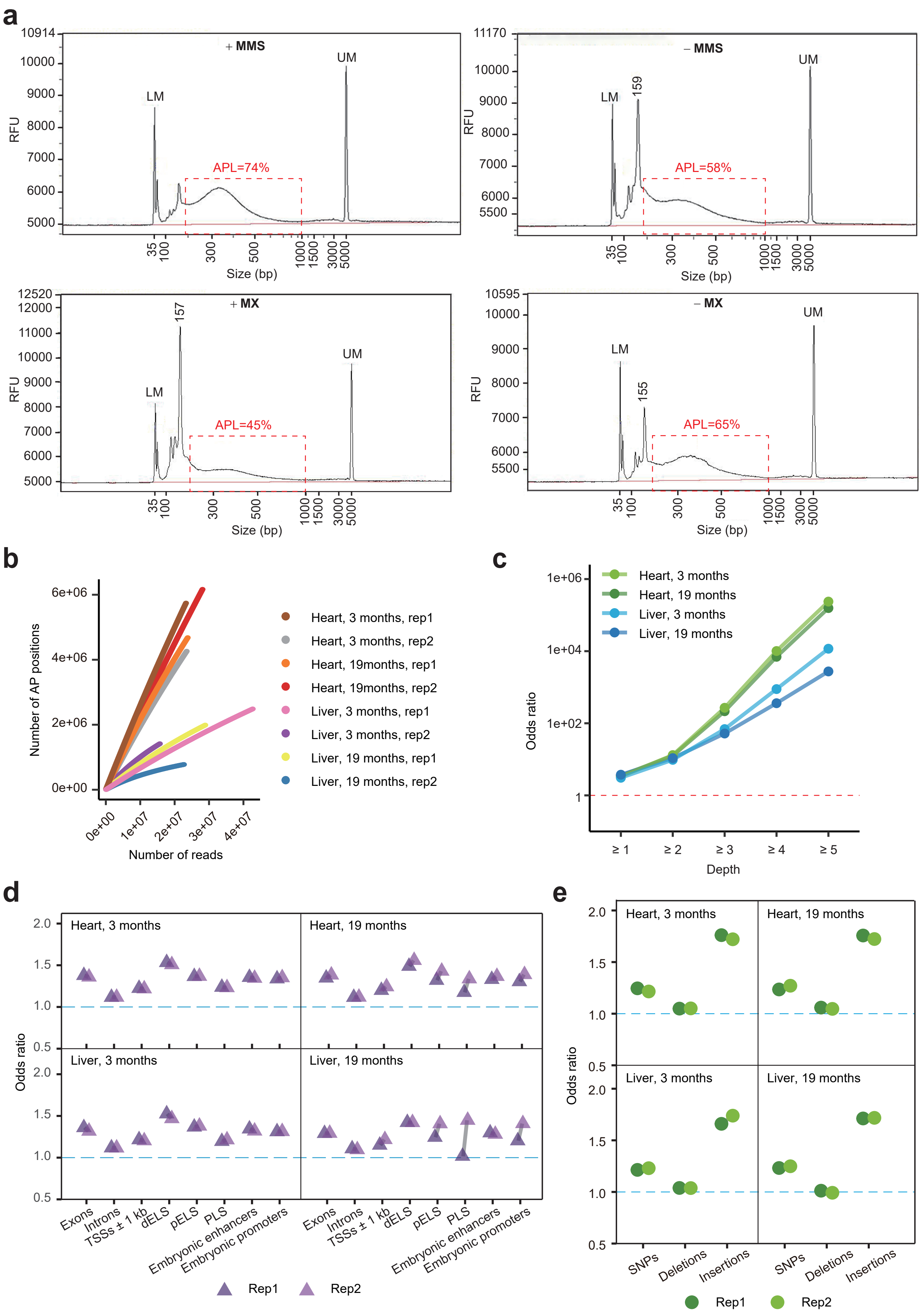

Supplementary Figure 3. Further validation of the performance of SSiNGLe-AP on mammalian genomes. a Representative DNA size distribution profiles of NGS libraries from MMS- or MX-treated and control samples of the HeLa cell line. APL represents the fraction of DNA with lengths larger than primer dimers in the libraries. The portions of the profiles representing the actual genomic material are boxed — the peaks to the left represent primer dimers. **b** The number of AP sites (unique genomic positions) identified from the given number of quality filtered NGS reads that were used as input in the analytical pipeline (Supplementary Fig. 1). **c** Distributions of the odds ratios of overlap of AP sites detected in technical replica 1 vs. those detected in replica 2 of the SSiNGLe-AP libraries made on heart and liver tissues from 3- and 19-month-old mice. The odds ratios of overlap are given for AP sites detected with the indicated read depths (≥ 1, ≥ 2 and so on). **d** Overlap between AP sites detected in each of two technical replicates and different classes of genomic elements. The dELS, pELS and PLS represent candidate cis-regulatory elements with distal enhancer-like, proximal enhancer-like and promoter signatures, respectively. **e** Overlap of AP sites in each of two technical replicates with different types of sequence variants. Source data are provided as a Source Data file.

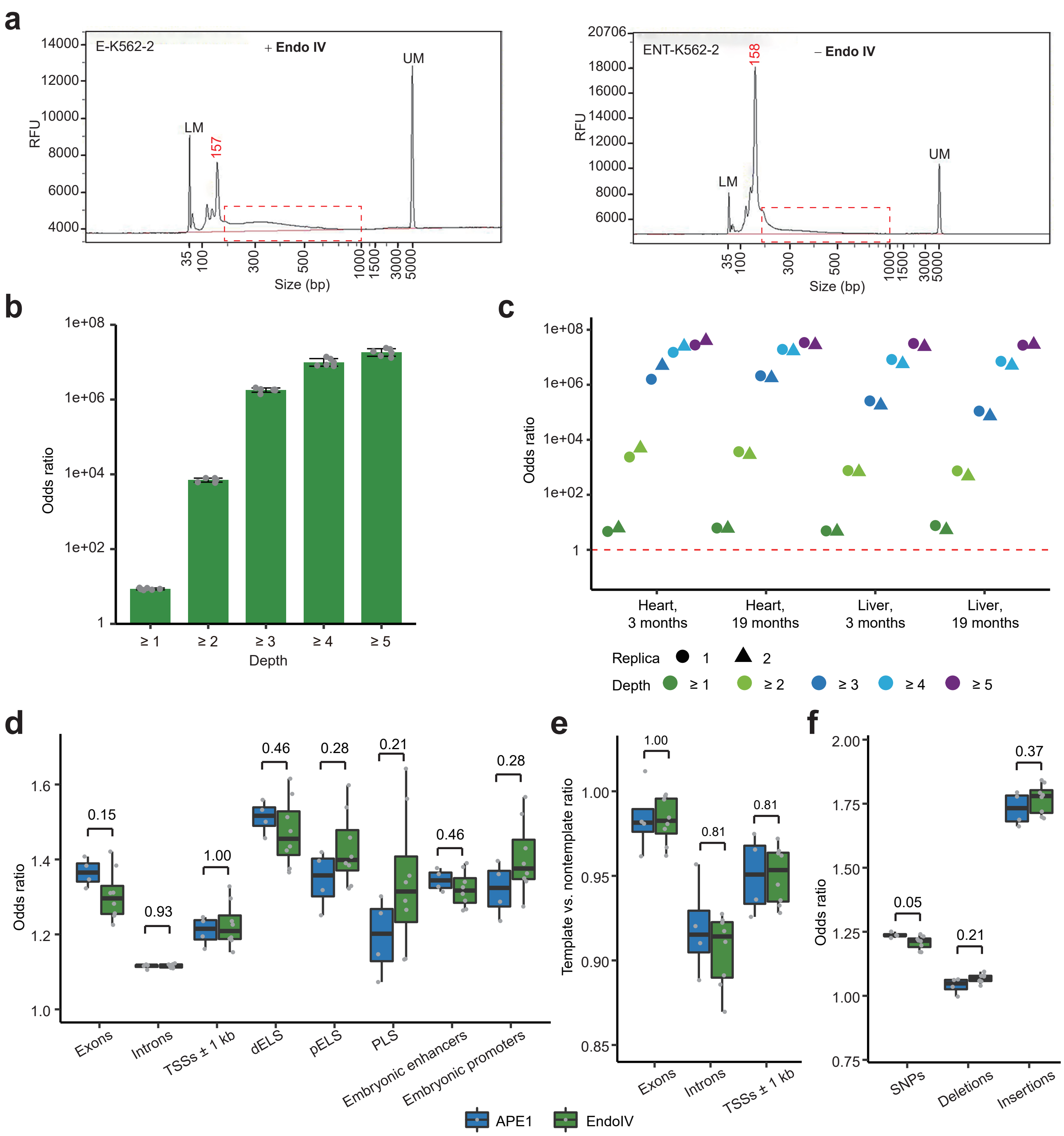

Supplementary Figure 4. Comparisons of the genomic landscapes of all AP sites detected using SSiNGLe-AP with either APE1 or Endo IV. a Representative DNA size distribution profiles of the NGS libraries made using SSiNGLe-AP with and without Endonuclease IV (Endo IV) enzyme. The portions of the profiles representing the actual genomic material are boxed — the peaks to the left represent primer dimers. **b** Bar plots of average odds ratios of overlap of AP sites detected in the SSiNGLe-AP libraries made with APE1 vs. AP sites detected in the libraries made with Endo IV in the K562 cell line. Data are presented as mean values +/- SD based on 8 comparisons. **c** Dot plot of the odds ratio of overlap of AP sites detected in the SSiNGLe-AP libraries made with APE1 vs. AP sites detected in two separate technical replicas of libraries made with Endo IV from the heart and liver tissues from 3- and 19-month-old mice. The odds ratios of overlap are given for AP sites detected with the indicated read depths (≥ 1, ≥ 2 and so on) (**b, c**). **d** Overlap between AP sites and different classes of genomic elements. The dELS, pELS and PLS represent candidate cis-regulatory elements with distal enhancer-like, proximal enhancer-like and promoter signatures, respectively. **e** Distribution of the template vs. nontemplate ratios for the AP sites found in the indicated elements. **f** Overlap of AP sites with different types of sequence variants. **d–f** Samples are the same as mentioned in **c**. Box plots indicate median (middle line), 25th, 75th percentile (box) and 1.5× interquartile range (whiskers) as well as each individual data (single points).The numbers above connecting lines are raw *p*-values for the indicated comparisons calculated by the two-sided Wilcoxon rank-sum test. Source data are provided as a Source Data file.

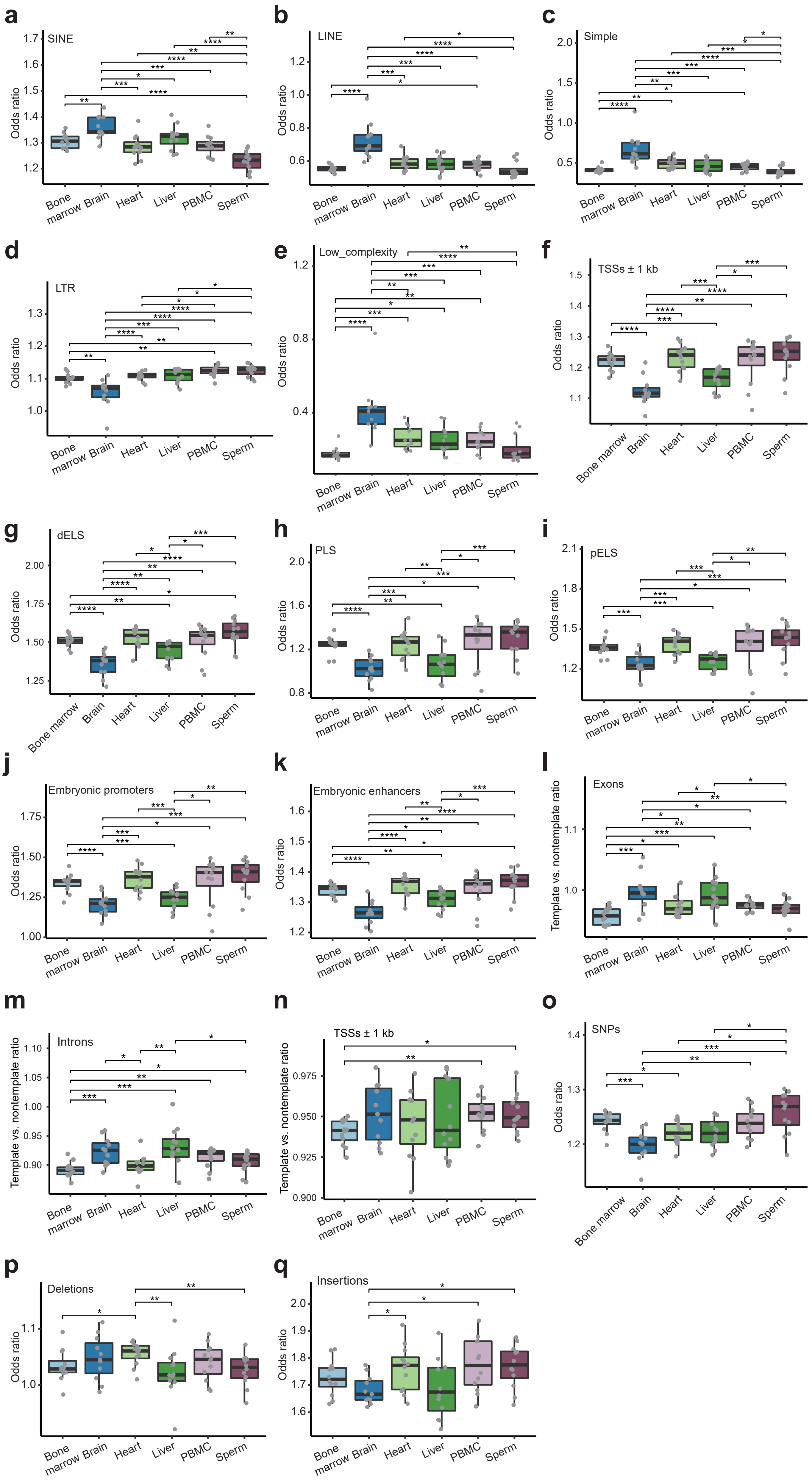

Supplementary Figure 5. Overlap of all AP sites with various genomic features in different mouse tissue types. a–**e** Overlap between AP sites and different repeat classes: SINE (**a**), LINE (**b**), simple (**c**), LTR (**d**) and low complexity (**e**). **f**–**k** Overlap between AP sites and different candidate cis-regulatory elements was as follows: regions around (±1 kb) TSSs (**f**), dELS (distal enhancer-like signatures, **g**), PLS (promoter-like signatures, **h**), pELS (proximal enhancer-like signatures, **i**), embryonic promotors (**j**) and embryonic enhancers (**k**). **l**–**n** Box plots of the distribution of template vs. nontemplate ratios for the AP sites found in the indicated elements. **o**–**q** Overlap of AP sites and the following different types of sequence variants: SNPs (**o**), deletions (**p**) and insertions (**q**). **a**–**q** Box plots indicate median (middle line), 25th, 75th percentile (box) and 1.5× interquartile range (whiskers) as well as each individual data (single points) based on 12 (3 biological replicates of 4 age groups) biologically independent samples per tissue type with the exception of brain represented by 11 samples. Asterisks above connecting lines indicate the significance of difference between the indicated pairs of tissues as represented by raw *p*-values of ≤ 0.05 (*), ≤ 0.01 (**), ≤ 0.001 (***) and ≤ 0.0001 (****) that were calculated by the two-sided Wilcoxon rank-sum test. Source data are provided as a Source Data file.

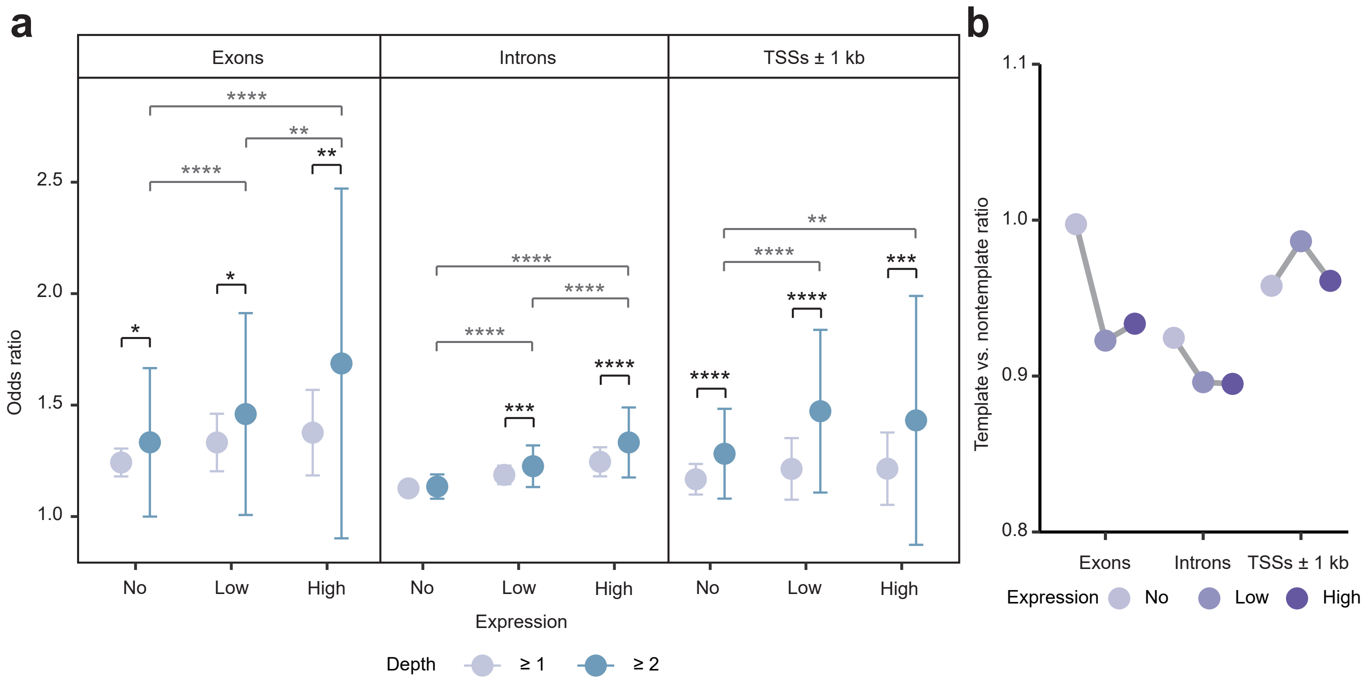

Supplementary Figure 6. Overlap between the sample-level AP site hotspots that were detected at read depth ≥ 2 and exons, introns and TSS flanking regions of genes stratified by expression level. a Distribution of the average odds ratios of overlap between sample-level hotspots and the indicated elements for each gene expression category. The error bars represent standard deviations. Data are presented as mean values +/- SD based on 71 mouse samples across all tissue types. The asterisks above connecting lines indicate significance of differences between the indicated pairs of gene expression categories, or between all AP sites and hotspots, as represented by raw *p*-values of ≤ 0.05 (*), ≤ 0.01 (**), ≤ 0.001 (***) and ≤ 0.0001 (****) as calculated by the two-sided Wilcoxon signed rank test. **b** Template vs. nontemplate ratios for the indicated elements were calculated for each gene expression category. Due to the low number of hotspots in exons and TSS flanking regions, the hotspots were summed up for each strand across the 71 samples and then used to calculate the corresponding ratios (Supplementary Data 13). As in the case of all AP sites, the hotspots were also more enriched on the nontemplate strands of exons and introns with increased expression. Note that the number of hotspots in the exons of highly expressed genes was small, and thus, the ratio for this category is less reliable, which likely explains the lack of a further drop in the ratio in this category. **a, b** All known genes were classified into three categories — nonexpressed genes with TPM ≤ 1, low expressed genes with 1 < TPM ≤ 10 and highly expressed genes with TPM > 10. Source data are provided as a Source Data file.

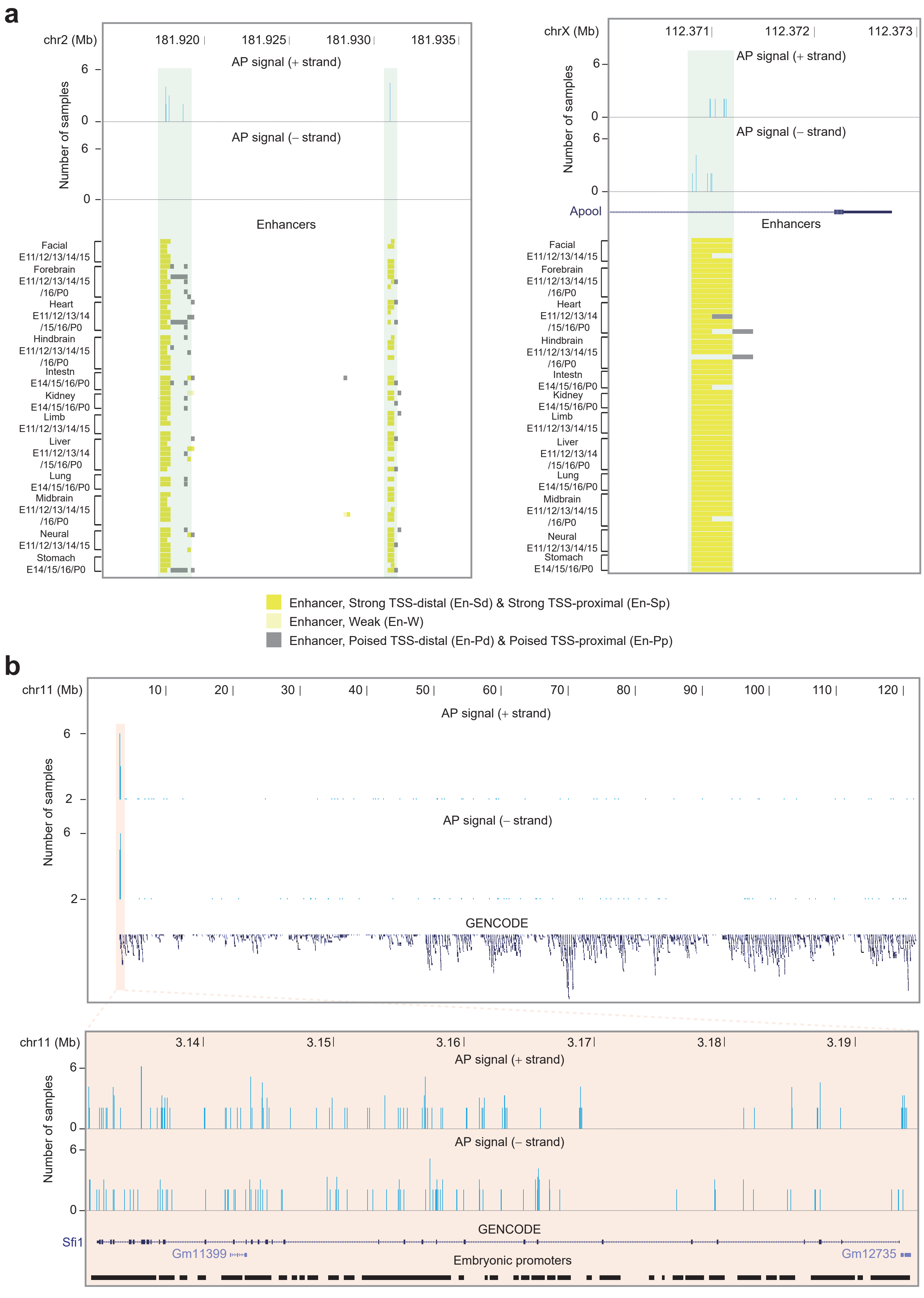

Supplementary Figure 7. Representative genome browser views of the sample-shared AP site hotspots in the mouse genome. a Two different genomic regions containing sample-shared hotspots mapped to constitutive embryonic enhancers found in the indicated embryonic tissues and/or times of development. **b** Whole chromosome view of the distribution of sample-shared hotspots on chr11 with a zoomed-in view of the peaks of hotspots mapping to the *Sfi1* locus. The distribution of hotspots (*Y*-axes) is represented by the numbers of samples where the hotspots were found (**a, b**). Source data are provided as a Source Data file.

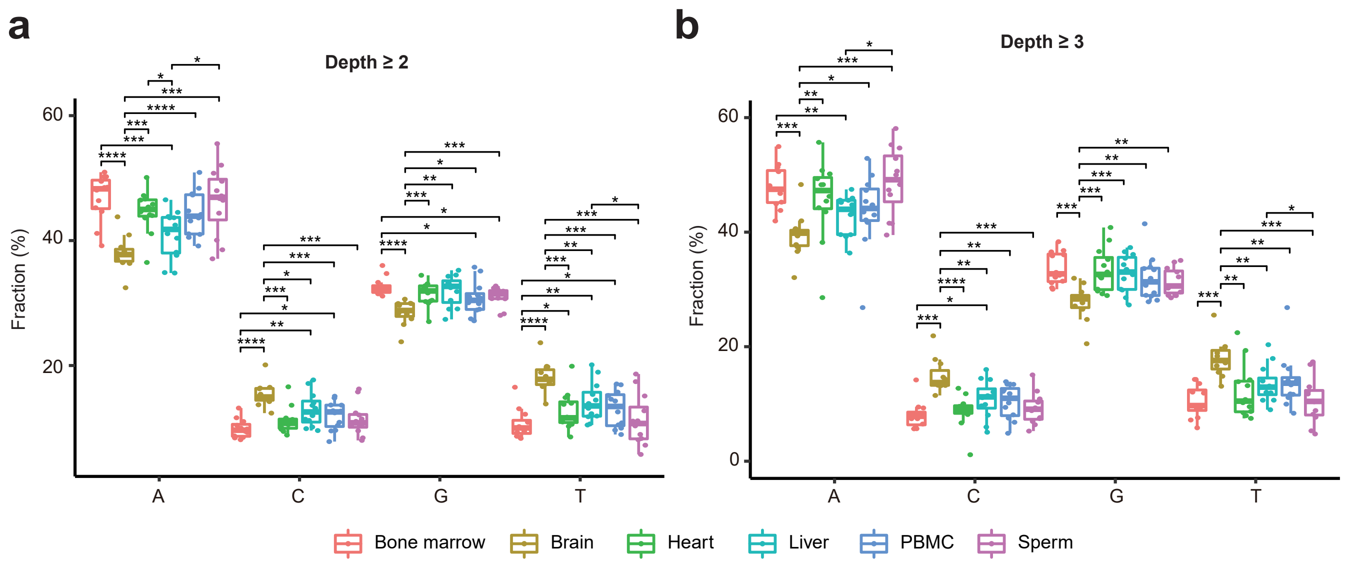

Supplementary Figure 8. Nucleotide preference of the sample-level AP site hotspots. a Fraction of each nucleotide for hotspots with read depth ≥ 2 in different tissue types. **b** Fraction of each nucleotide for hotspots with read depth ≥ 3 in different tissue types. **a**, **b** Box plots indicate median (middle line), 25th, 75th percentile (box) and 1.5× interquartile range (whiskers) as well as each individual data (single points) based on 12 (3 biological replicates of 4 age groups) biologically independent samples per tissue type with the exception of brain represented by 11 samples. Asterisks above connecting lines indicate significance of difference between the indicated pairs of tissues as represented by raw *p*-values of ≤ 0.05 (*), ≤ 0.01 (**), ≤ 0.001 (***) and ≤ 0.0001 (****) calculated by the two-sided Wilcoxon rank-sum test. Source data are provided as a Source Data file.
