# Supplementary material for: Complex genomic patterns of abasic sites in mammalian DNA revealed by a high-resolution SSiNGLe-AP method
Source: Nat Commun. 2022 Oct 5;13:5868. doi: 10.1038/s41467-022-33594-1 (PMC9534904; doi:10.1038/s41467-022-33594-1)
Supplement: Supplementary file 2 — Description of additional Supplementary File [file 41467_2022_33594_MOESM2_ESM.pdf]

Descriptions of Additional Supplementary Files

Supplementary Data 1. Characteristics of the human K562 samples containing spike-ins.

Supplementary Data 2. Relative depths of mapped AP sites along the length of sequences from the 3 combined spike-ins.

Supplementary Data 3. Nucleotide distribution of all AP sites and hotspots in the spike-ins.

Supplementary Data 4. Normalized relative depths of AP sites on "+" strand of the spike-in #1 shown in Fig. 2e from 3 independent libraries containing either positive ("P-A") and negative ("N-A") spike-ins.

Supplementary Data 5. The abundances and distributions of AP sites and hotspots detected in the MMS and MX experiments.

Supplementary Data 6. Analysis results of the deep-sequenced SSiNGLe-AP libraries and the corresponding unblocked controls.

Supplementary Data 7. Overlap of AP sites within each of the 4 pairs of technical replicas of the deep-sequenced samples.

Supplementary Data 8. Overlap of AP sites detected by SSiNGLe-AP using either Endo IV or APE1.

Supplementary Data 9. Characteristics of the 72 mouse SSiNGLe-AP samples.

Supplementary Data 10. Characteristics of the 71 mouse SSB samples.

Supplementary Data 11. Overlap of AP sites with various repeat classes in each of the 71 mouse samples.

Supplementary Data 12. Overlap of AP sites with various genomic features in the mouse samples generated using SSiNGLe-AP with either APE1 or Endo IV.

Supplementary Data 13. Distribution of all AP sites and sample-level hotspots found with depth ≥ 2 in exons, introns and the regions flanking TSSs of genes in the 3 expression categories.

Supplementary Data 14. TPM of the *Apex1* gene in each of the 71 mouse samples.

Supplementary Data 15. The fraction of sample-level hotspots found in the real and simulated datasets at different read depths.

Supplementary Data 16. Overlap of sample-level hotspots with various genomic features.

Supplementary Data 17. Number of sample-shared hotspots and their overlap with different genomic features.

Supplementary Data 18. Genomic coordinates of hotspots shared by at least 2 samples.

Supplementary Data 19. Fraction of each nucleotide for either all AP sites or sample-level hotspots with different depths in each sample.

Supplementary Data 20. Fraction of each nucleotide for all sample-level hotspots found with depth $\geq$ 2 shared by the indicated numbers of samples.

Supplementary Data 21. Correlation between age and various features of AP sites found with different depths in different tissues.

Supplementary Data 22. The AP site signals on the plus (L) or minus (H) strand of the mitochondrial genome in each of the 71 mouse samples.
